# Supplementary material for: Segmenting Increasing- and High-Risk Alcohol Drinkers by Motives and Occasions: Implications for Targeted Interventions
Source: Nutrients. 2025 Nov 28;17(23):3745. doi: 10.3390/nu17233745 (PMC12694210; doi:10.3390/nu17233745)
Supplement: Supplementary file 1 [file nutrients-17-03745-s001.zip › nutrients-3988391-supplementary.pdf]

Supplementary Material

Figure S1 - Complete Drinkaware Monitor Questionnaire 2023

Drinkaware Monitor 2023

*Question type: **Text***

In this survey you are invited to take part in a study being run by YouGov in partnership with charity and academic researchers. Before you decide, please take time to read this information page.

What is the aim of the study?
In this survey, we'll ask you a range of questions about lifestyle choices and health, with a particular interest in your drinking habits and attitudes towards alcohol in general. Even if you never drink alcohol, we're still very interested in hearing your opinions.

What topics are being covered?
We are going to ask you questions about your attitudes and behaviour regarding alcohol. The survey will also cover sensitive topics such as needling and drink spiking. Please remember that all responses collected are anonymous and you won't be identifiable from your answers. If you would prefer not to answer a question, please select the 'Prefer not to say' option.

What types of participants are being sought?
We are seeking the views and experiences of people aged 18 to 85 from across the UK.

How long will it take?
It should take around 20 minutes and you will receive 150 points for participating.

Can I change my mind and withdraw from the project?
Participation is voluntary so you can choose to opt out at any time by closing your browser. Only fully completed survey responses will be used.

What data or information will be collected and what use will be made of it?
The research team will have access to responses to the survey but will not be able to trace responses back to individuals, so participants can be assured of confidentiality and anonymity. Anonymised data will be retained for at least 10 years in secure storage. It may be shared with other researchers for independent analysis and handled in accordance with relevant ethical codes of conduct.

What if you have any questions?
If you have any questions or would like to discuss the study further, either now or in the future, feel free to contact:

Question type: ***PdI***
#Question display logic:
if pdl.profile_work_stat.last > days(100) and updated

[profile_work_stat] Which of these applies to you?

<1>	Working full time (30 or more hours per week)
<2>	Working part time (8-29 hours a week)
<3>	Working part time (Less than 8 hours a week)
<4>	Full time student
<5>	Retired
<6>	Unemployed
<7>	Not working
<8>	Other

Question type: ***PdI***
#Question display logic:
if profile_work_stat in [1,2,3] and pdl.employee_status.last > days(100) and updated

[employee_status] Are you employed by someone else, or are you self-employed? If both are true, then please think about the one that occupies more of your time.

<1>	Employee
<2>	Self-employed

Question type: ***Single***

[S1] At birth were you described as:

A later question gives the option to tell us if your gender is different from your sex registered at birth, and, if different, to record your gender.

We are asking this question because the effects of alcohol consumption, and some other health matters covered in this survey, are affected by your sex.

<1>	Male
<2>	Female
<3>	Intersex
<97>	Prefer not to say

*Question type: **Single***

[S2] Is the gender you identify with the same as your sex registered at birth?

<1>	Yes
<2>	No

*Question type: **Open***
#any
#Question display logic:
If [S2] - No is selected [if S2 == 2]

[S2a] How would you describe your current gender identity?

Prefer not to say

*Question type: **Single***

[S3] How would you describe your sexual orientation?

<1>	Bisexual
<2>	Heterosexual
<3>	Queer
<4>	Pansexual
<5>	Unsure
<6>	Lesbian
<7>	Asexual
<8>	Gay
<98>	Another way (open [s3_other]) [open] please specify
<99>	Prefer not to say

*Question type: **Text***

Moving on...

*Question type: **Single***

[A1] How often do you have a drink containing alcohol?

<1>	6 or more times a week
<2>	4 to 5 times a week
<3>	2 to 3 times a week
<4>	Once a week
<5>	2-3 times a month
<6>	Once a month
<7>	Once every couple of months
<8>	Once or twice a year
<9>	Less often
<99 fixed xor>	Never

Base: All non-drinkers
*Question type: **Single***
#Question display logic:
If [A1] - Never is selected [if A1 == 99]

[A1a] Which, if any, of the following best applies to you?

<1>	I used to drink alcohol but have given up
<2>	I have never drunk alcohol
<95>	Other (open [A1a_other]) [open] please specify

Base: Those who have given up alcohol
*Question type: **Multiple***
#row order: randomize #Columns: 2
#Question display logic:
If [A1a] - I used to drink alcohol but have given up is selected [if A1a == 1]

[A1b] What were your reasons for giving up alcohol? Please select all that apply.

<1>	Religious reasons	<9>	Alcohol wasn’t fitting into my lifestyle anymore
<2>	Don’t like the taste of alcohol	<10>	Don’t like the physical effects it has on me
<3>	Don’t like the behavioural effects it has on me	<11>	To perform better at work
<4>	My partner/spouse doesn’t drink	<12>	Because of pregnancy
<5>	I felt my drinking was problematic	<13>	Because of family and childcare responsibilities
<6>	To lose weight	<95 fixed>	Other (open [A1b_other]) [open] please specify
<7>	To improve my health generally	<98 fixed xor>	Can’t remember

<8>	I was advised to by a healthcare professional	<97 fixed xor>	Prefer not to say

Base: All non-drinkers
*Question type: **Grid***
#row order: randomize
#Question display logic:
If [A1] - Never is selected [if A1 == 99]

[NEW_P1] Thinking about the last 12 months, how often, if at all, have you experienced the following:

-[NEW_P1_1]	Pressure to drink alcoholic drinks even if others know that you don't drink
-[NEW_P1_2]	Being asked to explain or justify why you aren't drinking alcohol
-[NEW_P1_3]	Not having a non-alcoholic alternative to alcoholic drinks available
-[NEW_P1_4]	Deciding not to attend events because they will serve alcohol
-[NEW_P1_5]	Trying to hide that you do not drink alcohol from others
-[NEW_P1_6]	Feeling like an outsider in a situation where others are drinking alcohol
-[NEW_P1_7]	Feeling that others are avoiding you because you do not drink alcohol
-[NEW_P1_8]	Ending up in an argument because you do not drink
<1>	Always
<2>	Often
<3>	Sometimes
<4>	Rarely
<5>	Never
<97>	Don't know

#Question display logic:
if drink==1

*Question type: **Text***

[IMAGE x1unit_jpg]

*Question type: **Single***

[Q6B] How many units of alcohol do you drink on a typical _day_ when drinking? Please use the above guidance to complete your answer.

<1>	1 or 2

<2>	3 or 4
<3>	5 or 6
<4>	7 to 9
<5>	10 or more

*Question type: **Text***

[IMAGE x1unit_jpg]

*Question type: **Single***

[Q6C] How often have you had $Units or more units on a single occasion in the last year? Again, please use the above guidance to complete your answer.

<1>	Never
<2>	Less than monthly
<3>	Monthly
<4>	Weekly
<5>	Daily or almost daily

*Question type: **Single***
#Question display logic:
if drink==1 and gryphon.interview_type=="test"

[WeekDay] which day of the week is it?

<1>	Sunday
<2>	Saturday
<3>	Friday
<4>	Thursday
<5>	Wednesday
<6>	Tuesday
<7>	Monday

*Question type: **Text***

[IMAGE x1unit_jpg]

*Question type: **Grid-Open***
#row order: rotate($qr)

[A3] Thinking about the seven days leading up to yesterday, please enter how many units you drank on each _day_. For days where you had no alcohol, please enter 0.

-[A3_1]	Sunday
-[A3_2]	Saturday
-[A3_3]	Friday

-[A3_4]	Thursday
-[A3_5]	Wednesday
-[A3_6]	Tuesday
-[A3_7]	Monday
<1>	Enter value below

Base: All drinkers
*Question type: **Single***
#Question display logic:
if drink==1

[A9] Is that a typical drinking week for you?

<1>	Yes, this is a typical drinking week
<2>	No, I have drunk less than I would typically
<3>	No, I have drunk more than I would typically
<4>	Not sure

Base: All drinkers
*Question type: **Grid***
#row order: randomize
#Question display logic:
if drink==1

[A4] The following are reasons that people sometimes give for drinking alcohol. Thinking of all the times you drink, how often would you say that you drink for the following reasons? Please tick the answer of your choice to each question.

-[A4_1]	Because it helps you to enjoy a party	-[A4_7]	To fit in with a group you like
-[A4_2]	Because it helps you when you feel depressed or nervous	-[A4_8]	Because it improves parties and celebrations
-[A4_3]	To cheer up when you are in a bad mood	-[A4_9]	To forget about your problems
-[A4_4]	Because you like the feeling	-[A4_10]	Because it's fun
-[A4_5]	To get a buzz	-[A4_11]	To be liked
-[A4_6]	Because it makes social gatherings more fun	-[A4_12]	So you won't feel left out

<1>	Almost never/never
<2>	Some of the time
<3>	Half of the time
<4>	Most of the time
<5>	Almost always/always

Base: All drinkers
*Question type: **Grid***

#Question display logic:
if drink==1

[A11] The following are reasons that people sometimes give for drinking alcohol. Thinking of all the times you drink, how often would you say that you drink for the following reasons? Please tick the answer of your choice to each question.

-[A11_1]	Because drinking is part of the fun with family or friends	-[A11_9]	To put you at ease with people
-[A11_2]	Because drinking adds a certain warmth to social occasions	-[A11_10]	Because it is satisfying to have a high-quality drink
-[A11_3]	To celebrate a special occasion with friends	-[A11_11]	Because it pairs well with food
-[A11_4]	To calm down when you are tense	-[A11_12]	Because there are certain products you particularly enjoy
-[A11_5]	To help you unwind	-[A11_13]	Because you like the taste
-[A11_6]	To make you more outgoing	-[A11_14]	Because it makes you happy
-[A11_7]	To overcome shyness	-[A11_15]	Because it gives you a pleasant feeling
-[A11_8]	Because you feel more self-confident and sure of yourself		

<1> Almost never/never
<2> Some of the time
<3> Half of the time
<4> Most of the time
<5> Almost always/always

Base: All drinkers
Question type: ***Dyngrid***
#row order: randomize
#Question display logic:
if drink==1

[A5_new_] How often, if at all, in the last year did you drink alcohol on occasions that are similar to the descriptions below? You can hover over some to see examples.

-[A5_new_1] Drinking at home alone
-[A5_new_2] A small number of drinks at home with people in my household
-[A5_new_3] Several drinks at home with people in my household
-[A5_new_4] Getting together at your or someone else's house
-[A5_new_5] Going out for a meal
-[A5_new_6] Evening or night out with friends
-[A5_new_8] Going out for a couple of drinks in the afternoon
-[A5_new_9] Drinking at events

<1>	Once a week or more
<2>	1-3 times a month
<3>	Once every 2-3 months or less often
<4>	Never
<5>	Not sure

Base: All drinkers
*Question type: **Scale***
#Question display logic:
if drink==1

[A_new_1] How important, if at all, is alcohol to your social life?

Range: Very important 1 ~ 5 Not at all important
Don't know / Prefer not to say

Base: All drinkers
*Question type: **Single***
#Question display logic:
if drink==1

[A7] Thinking about your alcohol consumption, how likely or not do you think it is that you will have increased health problems in the future if you continue to drink at your current level?

<1>	Very likely
<2>	Fairly likely
<3>	Not very likely
<4>	Not at all likely
<98>	Don’t know

Base: All drinkers
*Question type: **Grid***
#row order: randomize
#Question display logic:
if drink==1

[A10] If you are drinking, how often, if at all, do you drink the following?

-[A10_1]	Beer (e.g., lager, ale, stout)
-[A10_2]	Wine (e.g., red, white, rosé, sparkling)
-[A10_3]	Spirits (e.g., vodka, whiskey, gin, rum)
-[A10_4]	Prepared/ready to serve cocktails (including ‘alcopops’)
-[A10_5]	Ciders or Perry
<1>	Always
<2>	Often
<3>	Sometimes

<4>	Rarely
<5>	Never

Question type: ***Text***

Moving on...

Question type: ***Grid***
#row order: randomize
#Question display logic:
if drink==1

[A8] Here are some things people have said they do to moderate their drinking. Have you tried any of these?

-[A8_1]	Avoid always having alcohol in the house	-[A8_7]	Avoid drinking alcohol on a ‘school/work night’
-[A8_2]	Record how much I am drinking	-[A8_8]	Avoid being in a round of drinks
-[A8_3]	Drink a lower strength alcoholic drink	-[A8_9]	Drink within the guidelines
-[A8_4]	Alternate alcoholic drinks with soft drinks or water	-[A8_10]	Stay off alcohol for a fixed time period
-[A8_5]	Drink smaller glasses of wine or smaller bottles of beer	-[A8_11]	Take drink-free days during the week
-[A8_6]	Set myself a drinking limit e.g. just a glass/bottle	-[A8_12]	Drink non-alcoholic beer, wine or spirit substitutes

<1>	I have been doing this for a while
<2>	I started doing this recently (in the last two or three months)
<3>	I have done this in the past but I no longer do it
<4>	I am not doing this but would be willing to do so
<5>	I could never see myself doing this

#Question display logic:
if drink==1

Question type: ***Single***

[T1] How often during the last year have you found that you were not able to stop drinking once you had started?

<1>	Never
<2>	Less than monthly
<3>	Monthly
<4>	Weekly

<5> Daily or almost daily

*Question type: **Single***

[T2] How often during the last year have you failed to do what was normally expected from you because of your drinking?

<1> Never
<2> Less than monthly
<3> Monthly
<4> Weekly
<5> Daily or almost daily

*Question type: **Single***

[T3] How often during the last year have you needed an alcoholic drink in the morning to get yourself going after a heavy drinking session?

<1> Never
<2> Less than monthly
<3> Monthly
<4> Weekly
<5> Daily or almost daily

*Question type: **Single***

[T4] How often during the last year have you had a feeling of guilt or remorse after drinking?

<1> Never
<2> Less than monthly
<3> Monthly
<4> Weekly
<5> Daily or almost daily

*Question type: **Single***

[T5] How often during the last year have you been unable to remember what happened the night before because you had been drinking?

<1> Never
<2> Less than monthly
<3> Monthly
<4> Weekly
<5> Daily or almost daily

*Question type: **Single***

[T6] Have you or somebody else been injured as a result of your drinking?

<1>	No
<2>	Yes, but not in the last year
<3>	Yes, during the last year

Question type: ***Single***

[T7] Has a relative or friend, doctor or other health worker been concerned about your drinking or suggested that you cut down?

<1>	No
<2>	Yes, but not in the last year
<3>	Yes, during the last year

Base: All that have had someone else be concerned about their drinking or suggested they cut down
Question type: ***Multiple***
#row order: randomize
#Question display logic:
If [T7] - Yes, but not in the last year or Yes, during the last year, is selected [if T7 in [2,3]]

[T8a] What actions did your friend, relative, doctor or other health worker take to address their concerns about your drinking? Please select all that apply.

<1>	Made a comment about it
<2>	Spoke to me about it / had a conversation
<3>	Sought advice or support for me from a professional or helpline
<4>	Encouraged me to seek help or treatment
<95 fixed>	Other (open [T8a_other]) [open] please specify
<999 fixed xor>	Prefer not to say

Base: All that experienced an action due to a respondent's drinking
Question type: ***Single***
#Question display logic:
if T8a.has_any([1,2,3,4,95])

[T9] Did anything happen as a result of these actions? If yes, please explain what happened using as much detail as possible in the box.

<1>	Yes (open [T9_other]) [open] please specify
<2>	No
<3>	Don’t know

Base: All drinkers
Question type: ***Grid***
#row order: randomize
#Question display logic:
if drink==1

[T10] How comfortable, or uncomfortable, would you feel if the following people started a conversation with you if they had concerns about your drinking?

-[T10_1]	Partner or spouse
-[T10_2]	Family member
-[T10_3]	Friend
-[T10_6]	Your GP
<1>	Not at all comfortable
<2>	Not very uncomfortable
<3>	Fairly comfortable
<4>	Very comfortable
<5>	Don't know
<97>	Not applicable
<999>	Prefer not to say

Base: All drinkers
Question type: ***Scale***
#Question display logic:
if drink==1

[T11] Thinking about your alcohol consumption over the past 12 months, please place yourself on the following scale:

Range: I never worry about my alcohol consumption 1 ~ 5 I often worry about my alcohol consumption
Don't know / Prefer not to say

Base: All drinkers
Question type: ***Single***
#Question display logic:
if drink==1

[T12] In the last 12 months, have you tried to reduce your alcohol consumption?

<1>	Yes - I successfully reduced my alcohol consumption
<2>	Yes - I successfully reduced my alcohol consumption for a bit, but have returned to typical levels of drinking
<3>	Yes - I tried to reduce my alcohol consumption but wasn't successful
<4>	No

*Question type: **Text***

Moving on...

Base: All drinkers
*Question type: **Dyngrid***
#row order: randomize
#Question display logic:
if drink==1

[DV3] Thinking about the last 12 months, how often, if at all, have you experienced the following?

-[DV3_1]	Pressure to drink alcoholic drinks even if others know you aren't drinking
-[DV3_2]	Being asked to explain or justify why you aren't drinking alcohol
-[DV3_3]	Trying to hide that you are not drinking alcohol from others
-[DV3_4]	Feeling like an outsider in a situation where others are drinking alcohol
<1>	Always
<2>	Often
<3>	Sometimes
<4>	Rarely
<5>	Never
<6>	Don't know

Base: All
*Question type: **Single***

[DVNEW] In the past 12 months, how often if at all, have you gone to the pub?

<1>	Daily or almost daily
<2>	Weekly
<3>	Monthly
<4>	Less than monthly
<5>	Never

Base: All who go to the pub (not never)
*Question type: **Multiple***
#row order: randomize
#Question display logic:
If [DVNEW] - Daily or almost daily or Weekly or Monthly or Less than monthly, is selected [if DVNEW in [1,2,3,4]]

[DV4] Which, if any, of the following are the main reasons you go to the pub? Please choose all that apply.

<1>	To socialise	<9>	To go for a meal
<2>	It’s a convenient location to meet others	<10>	For a special occasion, e.g., a party/celebration
<3>	For the variety and quality of drinks available	<11>	During/after taking part in a certain activity, e.g., playing football, going on a walk
<4>	It’s one of the only things to do where I live	<12>	For events or entertainment at a pub, e.g., watch a band, quiz night
<5>	To support my local pub/community	<13>	Before/after going to events at another venue, e.g., live sports, music/comedy gig
<6>	To get out the house/for somewhere to go	<95 fixed>	Other (open [DV4_other]) [open] please specify
<7>	To kill some time, e.g., while waiting for a train	<97 fixed xor>	Don’t know
<8>	To get drunk		

*Question type: **Text***

Moving on...

Base: All who drink at home
*Question type: **Multiple***
#row order: randomize
#Question display logic:
if A5_new_1 in [1,2,3]

[P1] Which, if any, of the following are important to you when buying alcohol to drink at home? Please select all that apply.

<1>	Price (value for money)	<8>	Bottle/can/pack size
<2>	Brand or reputation	<9>	Social responsibility or ethical considerations (e.g., fair trade, sustainability)
<3>	Taste or flavour	<10>	Health-related factors (e.g., low calorie, low sugar)
<4>	Low alcohol content or strength (% ABV)	<95 fixed>	Other (open [P1_other]) [open] please specify

<5>	High alcohol content or strength (% ABV)	<11 fixed xor>	None of the above
<6>	Recommendations from friends or family	<12 fixed xor>	Don't know
<7>	Promotions or discounts		

Question type: ***Text***

The next questions are about how other people's drinking might have affected you. We understand that this is a sensitive subject, so please use the 'prefer not to say' options if you do not feel comfortable answering the question.

If you are concerned about your own or another person's alcohol consumption, please speak to your GP or contact Drinkline on 0300 123 1110 (Mon-Fri 9 am - 8 pm, weekends 11 am - 4 pm). If you are in Scotland, you can also contact Drinkline Scotland on 0800 7314 314. Or click here for Drinkaware alcohol support services information.

Or here for We are With You alcohol support services information.

Base: All
Question type: ***Single***

[CO_1] In the last 12 months, have you been concerned about someone else's drinking? This could include family members, friends, work colleagues, or other people you know.

<1>	Yes
<2>	No
<3>	Not sure
<99>	Prefer not to say

Base: Those concerned by someone else's drinking
Question type: ***Multiple***
#row order: randomize
#Question display logic:
If [CO_1] - Yes is selected [if CO_1 == 1]

[CO_2] Whose drinking have you been concerned by? Please tick all that apply.

<1>	Parent/Guardian
<2>	Child
<3>	Partner/ex-partner
<4>	Sibling
<5>	Friend

<6>	Co-worker
<95 fixed>	Other (open [CO_2_other]) [open] please specify
<97 fixed xor>	Prefer not to say

Base: Those concerned by someone else's drinking
*Question type: **Multiple***
#row order: randomize
#Question display logic:
If [CO_1] - Yes is selected [if CO_1 == 1]

[CO_4] Which, if any, of the following made you concerned about someone else's drinking? Please select all that apply.

<1>	The amount of alcohol they were drinking
<2>	The number of days a week they were drinking
<3>	How drunk they would get when drinking
<4>	Getting into regular arguments or conflicts while drinking
<5>	Not meeting their responsibilities or obligations due to drinking
<6>	Their physical or mental health getting worse due to drinking
<7>	Engaging in risky behaviour while under the influence of alcohol (e.g., driving)
<8>	Blackouts or memory loss
<9>	Changes in behaviour or mood when drinking alcohol
<10>	Relationship problems caused by alcohol use
<11>	Getting into financial difficulties
<12>	Negative impact on work or job performance due to alcohol use
<13>	Others being concerned about their drinking
<95 fixed>	Other (open [CO_4_other]) [open] please specify
<999 fixed xor>	Prefer not to say

Base: Those concerned by someone else's drinking
*Question type: **Multiple***
#row order: randomize
#Question display logic:
If [CO_1] - Yes is selected [if CO_1 == 1]

[CO_5] What actions, if any, have you taken to address your concerns about someone else's drinking? Please select all that apply.

<4>	I have made a comment about it to them
<1>	I have spoken to them about it
<2>	I have sought advice or support from a professional or helpline
<3>	I have encouraged them to seek help or treatment
<95 fixed>	Other (open [CO_5_other]) [open] please specify
<99 fixed xor>	N/A - I have not taken any specific actions
<999 fixed xor>	Prefer not to say

*Question type: **Single***
#Question display logic:
if CO_5.has_any([1,2,3,95])

[CO_6a] Did anything happen as a result of the action(s) you took? If yes, please explain what happened using as much detail as possible in the box.

<1>	Yes (open [CO_6_other]) [open] please specify
<2>	No
<3>	Don’t know
<999>	Prefer not to say

Base: All
*Question type: **Dyngrid***
#row order: randomize

[CO_7a] How comfortable, or uncomfortable, would you feel about starting a conversation with the following people if you had concerns about their drinking?

-[CO_7a_1]	Partner or spouse
-[CO_7a_2]	Family member (e.g., parent, sibling, child)
-[CO_7a_3]	Friend
<1>	Not at all comfortable
<2>	Not very uncomfortable
<3>	Fairly comfortable
<4>	Very comfortable
<5>	Don’t know
<97>	Not applicable

*Question type: **Text***

Moving on...

*Question type: **Open***
#any

[D2] In the box below, please list any illnesses or health conditions that you know can be caused by alcohol.

Not Sure

Base: All
*Question type: **Multiple***
#row order: randomize

[D3] Which, if any, of the following health conditions do you think are linked to alcohol consumption? Please tick all that apply.

<1>	Stroke	<7>	Mental health problems
<2>	Erectile dysfunction	<8>	Obesity
<3>	Back ache	<9>	Brittle bones
<4>	Liver disease	<10>	Cancer
<5>	Heart problems	<99 fixed xor>	None of these
<6>	High blood pressure	<98 fixed xor>	Don’t know

Base: Those that link alcohol to cancer
*Question type: **Multiple***
#row order: randomize
#Question display logic:
if D3.has_any([10])

[D4] You indicated alcohol is linked to cancer, which of the following types of cancer. Please tick all that apply.

<1>	Oropharynx
<2>	Larynx
<3>	Oesophagus
<4>	Liver
<5>	Colon
<6>	Rectum
<7>	Breast cancer
<8>	Pancreas
<9>	Prostate
<10>	Skin cancer
<97 fixed xor>	Don't know

Base: All
*Question type: **Scale***

[D5] Previous studies have shown that alcohol causes at least seven types of cancer, including cancer of the bowel, breast, mouth, throat (oesophagus, larynx, and pharynx) and liver cancer (Cancer Research UK).

What impact, if any, does this have on your perceptions of drinking?

Range: It has no impact on me 1 ~ 5 It makes me want to change my drinking habits
Don't know

*Question type: **Text***

Moving on...

Base: All
*Question type: **Single***

[QN1] Thinking about your finances, which of the following best reflects your position?

<1>	I cannot afford my essential costs, and often have to go without things like food and heating
<2>	I can only just afford my essential costs and often struggle to make ends meet
<3>	I can normally comfortably cover the essentials, but I do not often have money for luxuries
<4>	I am relatively comfortable financially
<5>	I am very comfortable financially
<999>	Prefer not to say

Base: All drinkers
*Question type: **Single***
#Question display logic:
if drink==1

[QN2] Thinking about the last 6 months, have you drank more or less alcohol as a result of the cost of living crisis, or has it stayed about the same?

<1>	Much more
<2>	A little more
<3>	It has stayed about the same
<4>	A little less
<5>	Much less
<96>	Don't know

Base: All
*Question type: **Single***

[QN3] Thinking about the last 6 months, have you spent more or less on alcohol as a result of the cost of living crisis, or has it stayed about the same?

<1>	Much more
<2>	A little more
<3>	It has stayed about the same
<4>	A little less
<5>	Much less
<96>	Don't know

*Question type: **Text***

Moving on...

Base: All drinkers
*Question type: **Dyngrid***
#row order: randomize
#Question display logic:
if drink==1

[AP4] For each of the following, how healthy or unhealthy would you rate the relationship with alcohol?

-[AP4_1]	Yourself
-[AP4_2]	Your friends/family
-[AP4_4]	$country
<1>	Very unhealthy
<2>	Unhealthy
<3>	Neither healthy nor unhealthy
<4>	Healthy
<5>	Very healthy
<97>	Don’t know

Base: All
*Question type: **Dyngrid***
#row order: randomize

[AP5] To what extent would you support or oppose the following?

-[AP5_1]	A ban on adverts about alcohol
-[AP5_2]	A ban on adverts about alcohol for under 18s
-[AP5_3]	Making alcohol less visible in supermarkets/shops
-[AP5_4]	Making health warnings clearly and consistently presented on alcohol packaging
-[AP5_5]	Having a minimum price for alcohol
-[AP5_6]	A ban on alcohol sponsorship of sport clubs, events or competitions
-[Ap5_7]	Ingredient and nutritional labelling (including calories) on alcohol packaging
<1>	Strongly support
<2>	Tend to support
<3>	Neither support nor oppose
<4>	Tend to oppose
<5>	Strongly oppose
<6>	Don’t know

Base: All
Question type: ***Scale***

[AP6] How often, if at all, do you see adverts for alcohol?

Range: I never see adverts for alcohol 1 ~ 5 I always see adverts for alcohol
Don't know

Base: Those who have ever seen alcohol marketing
Question type: ***Dyngrid***
#Question display logic:
if AP6_scale in [2,3,4,5]

[AP7] How often do you see/ hear alcohol marketing in the following places?

-[AP7_1]	TV/on demand/cinema/radio
-[AP7_2]	Shops and supermarkets
-[AP7_3]	Outdoors (billboards, public transport)
-[AP7_4]	Online (including social media)
<1>	Very often
<2>	Often
<3>	Sometimes
<4>	Rarely
<5>	Never

Base: All
Question type: ***Dyngrid***
#row order: randomize

[AP8] To what extent do you agree or disagree with the following statements? (If you do not personally drink, please think about these statements in general)

-[AP8_1]	It is OK to get drunk regularly if you don’t drink every day
-[AP8_2]	It is OK to get drunk if it isn’t impacting on anyone else
-[AP8_4]	People often encourage others to drink or drink more
-[AP8_5]	If someone is not getting drunk, regular drinking will not affect their health
-[AP8_6]	Our society is understanding of people with drinking problems
-[AP8_7]	People with drinking problems can reach out for support without being judged
-[AP8_8]	Drinking at home alone is a cause of worry
<1>	Strongly disagree
<2>	Disagree
<3>	Agree
<4>	Strongly agree
<5>	Don’t know

Base: All drinkers
*Question type: **Single***
#Question display logic:
if drink==1

[AP9] To what extent would you agree or disagree that you ‘drink responsibly’?

<1>	Strongly disagree
<2>	Disagree
<3>	Agree
<4>	Strongly agree
<5>	Don’t know

Base: All drinkers
*Question type: **Open***
#any
#Question display logic:
if drink==1

[AP10] To the best of your knowledge, when you hear, or see, the term ‘responsible drinking’, what does that mean to you? Please type your answer with as much detail as possible in the text box below.

Don't know / Prefer not to say

*Question type: **Text***

The following questions are about your personal health and wellbeing.

Base: All
*Question type: **Grid***
#row order: randomize

[MH_4] Over the last 2 weeks, how often have you been bothered by the following problems?

-[MH_4_1]	Little interest or pleasure in doing things
-[MH_4_2]	Feeling down, depressed, or hopeless
-[MH_4_3]	Feeling nervous, anxious, or on edge
-[MH_4_4]	Not being able to stop or control worrying
<1>	Not at all
<2>	Several days
<3>	More than half the days
<4>	Nearly every day
<5>	Prefer not to say

Base: All
*Question type: **Dyngrid***
#row order: randomize

[MH_5] The next question is about how you feel about different aspects of your life. For each one, tell me how often you feel that way.

How often, if ever, do you feel…

-[MH_5_1]	That you lack companionship
-[MH_5_2]	Left out
-[MH_5_3]	Isolated from others
-[MH_5_4]	Lonely
<1>	Often/Always
<2>	Some of the time
<3>	Occasionally
<4>	Hardly ever
<5>	Never

*Question type: **Text***

Moving on...

Base: All
*Question type: **Multiple***
#row order: randomize

[SA_5] An alcohol assessment includes questions on how often you drink alcohol and how many units you tend to drink on a typical day.

In which, if any, of the following have you been asked to complete an alcohol assessment? Please select all that apply. If you have never been asked to complete an alcohol assessment, please select the "not applicable" option.

<1>	GP surgery
<2>	Other healthcare setting
<3>	Workplace
<4>	Online website or app
<5>	Substance or addiction treatment centre
<6>	Community health fair or event
<95 fixed>	Other (open [SA_5_other]) [open] please specify
<98 fixed xor>	Not applicable - I have never been asked to complete an alcohol assessment
<99 fixed xor>	Can't remember

Base: All
*Question type: **Multiple***

[SA_3] Have you ever contacted a service or organisation for help or advice about alcohol, either on your own behalf or on behalf of someone close to you?

<1>	Yes – on my own behalf
<2>	Yes – on behalf of someone else
<3 xor>	No
<99 xor>	Prefer not to say

Base: All
*Question type: **Multiple***
#row order: randomize

[SA_4a_new] If you needed support or advice about alcohol **on your own behalf**, which, if any, of the following would you reach out to? Please select all that apply.

<1>	General Practitioner (GP)
<2>	Other health professional (e.g. specialist doctor, nurse etc.)
<3>	Counsellor/therapist
<4>	Alcohol charity/organisation
<5>	App/digital service
<6>	Online community support
<7>	In person support group (e.g. Alcoholics Anonymous)
<99 fixed xor>	None of these

*Question type: **Text***

The next few questions are about drink spiking. We understand that this may also be a sensitive topic so please select the "Prefer not to say" option if you would rather not answer the question.

Base: All
*Question type: **Single***

[DS_8] Do you think you have been a victim of drink spiking in the last year?

<1>	Yes
<2>	No
<98>	Not sure
<99>	Prefer not to say

*Question type: **Single***

[DS_9] 'Needling' is a new issue which has been reported recently. It is the act of covertly injecting someone with a substance, such as drugs of abuse or pharmaceutical compounds, without the person's prior knowledge or consent.

Do you think you have been a victim of needling in the last year?

<1> Yes
<2> No
<98> Not sure
<99> Prefer not to say

Base: Those who have been a victim of drink spiking
Question type: ***Multiple***
#row order: randomize
#Question display logic:
If [DS_8] - Yes is selected [if DS_8 == 1]

[DS_10_new] In which, if any, of the following did the incident(s) of drink spiking take place? Please select all the answers that apply.

<1> Club
<2> Bar
<3> Private home
<4> Social event
<5> University/ College
<6> Family event
<7> Work
<95 fixed> Other (open [DS_10_new_other]) [open] please specify
<98 fixed xor> Prefer not to say

Base: All
Question type: ***Single***

[DS_22] Do you think you know what to do in case you or someone you know has experienced drink spiking?

<1> Yes
<2> No
<97> Prefer not to say

Base: All those who would know what to do
Question type: ***Open***
#any
#Question display logic:
If [DS_22] - Yes is selected [if DS_22 == 1]

[DS_23] What would you do if you or someone you know experienced drink spiking?

Don't know / Prefer not to say

Base: Those who have been a victim of needling
Question type: ***Multiple***

#row order: randomize
#Question display logic:
If [DS_9] - Yes is selected [if DS_9 == 1]

[DS_11] In which, if any, of the following settings did the incident(s) of drink needling take place? Please select all that apply.

<1>	Club
<2>	Bar
<3>	Private home
<4>	Social event
<5>	University/ College
<6>	Family event
<7>	Work
<95 fixed>	Other (open [DS_11_open]) [open] please specify
<99 fixed xor>	Prefer not to say

Base: All
Question type: ***Single***

[DS_24] Do you think that you know what to do if you or someone you know has experienced needling?

<1>	Yes
<2>	No
<3>	I am not sure
<97>	Prefer not to say

Base: All those who would know what to do
Question type: ***Open***
#any
#Question display logic:
If [DS_24] - Yes is selected [if DS_24 == 1]

[DS_25] What would you do if you or someone you know has experienced drink needling?

Don't know / Prefer not to say

Question type: ***Text***

Thank you for completing this survey. If you have been affected by any of the issues discussed then we have listed several organisations below which you can turn to for help or support

Alcohol
If you are concerned about your own or another person’s alcohol consumption,

please speak to your GP or contact Drinkline on 0300 123 1110 (Mon-Fri 9 am - 8 pm, weekends 11 am - 4 pm) or click here for Drinkaware alcohol support services information. If you are in Scotland, you can also contact Drinkline Scotland on 0800 7314 314.
We are With You also provide free confidential support with alcohol, drugs or mental health. You can access their website here.

Rape and sexual assault
If you would like to get support after experiencing rape, sexual assault, sexual abuse or any type of sexual violence you can contact Rape Crisis in England and Wales here.

Please click here to get support if you are in Scotland and here to get support if you are in Northern Ireland.

Alternatively, you can contact Victim Support by calling 0808 1689 111 or visiting their website here.

Samaritans
Finally, if you would just like to talk to someone about whatever you are going through, you can call Samaritans for free on 116 123 or visit their website here

Base: All
*Question type: **Single***

[caring_responsibilities] Do you currently look after, or give any help or support to anyone because they have long-term physical or mental health conditions or illnesses?

<1>	No
<2>	Yes, 9 hours a week or less
<3>	Yes, 10 to 19 hours a week
<4>	Yes, 20 to 34 hours a week
<5>	Yes, 35 to 49 hours a week
<6>	Yes, 50 or more hours a week
<97>	Don't know
<999>	Prefer not to say

Base: All
*Question type: **Multiple***

[health_conditions] Which, if any, of the following health conditions have you been diagnosed with? Please select all that apply.'

<1>	Depression	<7>	Musculoskeletal conditions (e.g. arthritis, fibromyalgia)

<2>	Other mental health conditions (e.g. OCD)	<8>	Hypothyroidism
<3>	Respiratory conditions (e.g. Asthma)	<9>	Cancer
<4>	Diabetes	<10>	Neurological conditions (e.g. Epilepsy, Dementia, Parkinson’s disease)
<5>	Cardiovascular conditions (e.g. high blood pressure, COPD, heart disease)	<99 fixed xor>	None of these
<6>	Chronic Kidney Disease	<999 fixed xor>	Prefer not to say

*Question type: **Text***

Finally, some demographic questions for our records...

*Question type: **Pdl***
#Question display logic:
if not pdl.ethnicity_new and updated

[ethnicity_new] What ethnic group best describes you? Please select one option only. (We ask the question in this way so that it is consistent with Census definitions.)

<1>	English / Welsh / Scottish / Northern Irish / British	<11>	Bangladeshi
<2>	Irish	<12>	Chinese
<3>	Gypsy or Irish Traveller	<13>	Any other Asian background
<4>	Any other White background	<14>	African
<5>	White and Black Caribbean	<15>	Caribbean
<6>	White and Black African	<16>	Any other Black / African / Caribbean background
<7>	White and Asian	<17>	Arab
<8>	Any other Mixed / Multiple ethnic background	<18 fixed>	Any other ethnic group
<9>	Indian	<19 fixed>	Prefer not to say
<10>	Pakistani		

*Question type: **Pdl***
#Question display logic:
if pdl.profile_marital.last > months(6) and updated

[profile_marital] What is your current marital or relationship status?

<7>	Divorced
<2>	In a civil partnership
<5>	In a relationship, but not living together
<4>	Living with a partner but neither married nor in a civil partnership
<1>	Married
<3>	Separated but still legally married or in a civil partnership
<6>	Single
<8>	Widowed

Question type: ***PdI***
#Question display logic:
if pdl.profile_household_size.last > months(6) and updated

[profile_household_size] How many people, including yourself, are there in your household? Please include both adults and children.

<1>	1
<2>	2
<3>	3
<4>	4
<5>	5
<6>	6
<7>	7
<8>	8 or more
<9>	Don't know
<10>	Prefer not to say

Question type: ***PdI***
#Question display logic:
if pdl.parent.last > months(6) and updated

[parent] Are you a parent or guardian? Please select all that apply

<1>	Yes, of at least one child younger than 18 years old
<2>	Yes, of at least one child 18 years old or older
<97 xor>	No, I am neither a parent or guardian
<98 xor>	Don't know/Prefer not to say

Question type: ***PdI***
#Question display logic:
if pdl.profile_education_level.last > months(6) and updated

[profile_education_level] What is the highest educational or work-related qualification you have?

<1>	No formal qualifications	<11>	GCE A level or Higher Certificate
<2>	Youth training certificate/skillseekers	<12>	Scottish Higher Certificate
<3>	Recognised trade apprenticeship completed	<13>	Nursing qualification (e.g. SEN, SRN, SCM, RGN)
<4>	Clerical and commercial	<14>	Teaching qualification (not degree)
<5>	City & Guilds certificate	<15>	University diploma
<6>	City & Guilds certificate - advanced	<16>	University or CNAA first degree (e.g. BA, B.Sc, B.Ed)
<7>	ONC	<17>	University or CNAA higher degree (e.g. M.Sc, Ph.D)
<8>	CSE grades 2-5	<18>	Other technical, professional or higher qualification
<9>	CSE grade 1, GCE O level, GCSE, School Certificate	<19>	Don't know
<10>	Scottish Ordinary/ Lower Certificate	<20>	Prefer not to say

*Question type: **Pdl***
#Question display logic:
if pdl.profile_household_children.last > months (12) and updated

[profile_household_children] How many of the people in your household are under 18?

<1>	0
<2>	1
<3>	2
<4>	3
<5>	4
<6>	5 or more
<8>	Don't know
<9>	Prefer not to say

*Question type: **Pdl***
#Question display logic:
if pdl.disability.last > months(6) and updated

[disability] Are your day-to-day activities limited because of a health problem or disability which has lasted, or is expected to last, at least 12 months?

<1>	Yes, limited a lot
<2>	Yes, limited a little

<3> No

*Question type: **Pdl***
#Question display logic:
if pdl.smoker.last > months(6) and updated

[smoker] Which, if any, of the following statements BEST applies to you, when it comes to smoking?

<1>	I smoke every day
<2>	I smoke but I don't smoke every day
<3>	I used to smoke but I have given up now
<4>	I have never smoked
<99>	Prefer not to say

*Question type: **Pdl***
#Question display logic:
if pdl.birthplace_UK.last > months(12) and updated

[birthplace_UK] What is your place of birth?

<1>	England
<2>	Scotland
<3>	Wales
<4>	Northern Ireland
<5>	Outside the UK

*Question type: **Pdl***
#Question display logic:
if pdl.profile_religion_stat.last > months (12) and updated

[profile_religion_stat] Do you regard yourself as belonging to any particular religion?

<1>	Yes
<2>	No
<3>	Don't know
<4>	Prefer not to say

*Question type: **Pdl***
#Question display logic:
if pdl.profile_religion_denom.last > months (12) and profile_religion_stat==1 and updated

[profile_religion_denom] If so, which denomination?

<1>	None	<9>	Brethren

<2>	Church of England/Anglican/Episcopal	<10>	Jewish
<3>	Roman Catholic	<11>	Hindu
<4>	Presbyterian/Church of Scotland	<12>	Islam/Muslim
<5>	Methodist	<13>	Sikh
<6>	Baptist	<14>	Buddhist
<7>	United Reformed Church	<15>	Other
<8>	Free Presbyterian	<16>	Prefer not to say

Table S2 - Complete two-proportion z-test results for cluster-benchmark comparisons

Results for each question analysed are presented as weighted proportions for all clusters versus the benchmark (all increasing/high-risk drinkers). Differences are presented with 95% confidence intervals. Significance is shown with *p*-values rounded to three decimal places or as <0.001, with Benjamini-Hochberg false discovery rate (FDR) adjusted p-values reported. For each question where significance was found, adjusted *p* < 0.05 are bolded and highlighted in green.

Answer	Cluster	Cluster Proportion	Benchmark proportion	Difference	95% CI Low	95% CI Upper	*p*_value	Adjusted p-value (FDR)	Significant (FDR < 0.05)	Test Statistic	Direction
East_of_England	1	0.08365779	0.07627783	0.00737996	-0.016745091	0.031505011	0.538642519	0.76631616	FALSE	0.614867059	Cluster higher
East_of_England	2	0.073463964	0.07627783	-0.002813866	-0.023527777	0.017900045	0.791831505	0.903080559	FALSE	-0.263933054	Cluster lower
East_of_England	3	0.053871971	0.07627783	-0.022405859	-0.04846154	0.003649822	0.135945662	0.390843778	FALSE	-1.491060309	Cluster lower
East_of_England	4	0.080791836	0.07627783	0.004514006	-0.02735123	0.036379242	0.776900193	0.898277669	FALSE	0.283360907	Cluster higher
East_of_England	5	0.057669361	0.07627783	-0.018608469	-0.058620367	0.021403429	0.416972865	0.753690746	FALSE	-0.811684196	Cluster lower
East_of_England	6	0.102773984	0.07627783	0.026496154	-0.012675512	0.06566782	0.139117442	0.391800142	FALSE	1.479085588	Cluster higher
IMD_decile_2020_1_to_3	1	0.262767484	0.305302043	-0.04253456	-0.081565151	-0.003503968	0.037776373	0.155675645	FALSE	-2.077272967	Cluster lower
IMD_decile_2020_1_to_3	2	0.322191255	0.305302043	0.016889212	-0.019921338	0.053699762	0.365034182	0.740804664	FALSE	0.905814241	Cluster higher
IMD_decile_2020_1_to_3	3	0.317000987	0.305302043	0.011698944	-0.040732699	0.064130587	0.659405587	0.810122223	FALSE	0.44073399	Cluster higher
IMD_decile_2020_1_to_3	4	0.289578832	0.305302043	-0.015723211	-0.069004657	0.037558235	0.567626282	0.788195419	FALSE	-0.571550878	Cluster lower
IMD_decile_2020_1_to_3	5	0.295163901	0.305302043	-0.010138142	-0.087854257	0.067577974	0.799894852	0.904799095	FALSE	-0.253483187	Cluster lower
IMD_decile_2020_1_to_3	6	0.36578589	0.305302043	0.060483847	-0.002079197	0.123046891	0.049504996	0.186609693	FALSE	1.964216447	Cluster higher
IMD_decile_2020_4_to_7	1	0.405817205	0.389752646	0.016064559	-0.027014276	0.059143394	0.463047223	0.753690746	FALSE	0.733838419	Cluster higher
IMD_decile_2020_4_to_7	2	0.37350348	0.389752646	-0.016249166	-0.054567101	0.022068769	0.407873298	0.753690746	FALSE	-0.827641959	Cluster lower
IMD_decile_2020_4_to_7	3	0.404692188	0.389752646	0.014939543	-0.040394032	0.070273117	0.594918899	0.797062065	FALSE	0.5317215	Cluster higher
IMD_decile_2020_4_to_7	4	0.40984509	0.389752646	0.020092444	-0.037526677	0.077711565	0.491537443	0.753690746	FALSE	0.687865565	Cluster higher
IMD_decile_2020_4_to_7	5	0.419492461	0.389752646	0.029739815	-0.054246108	0.113725738	0.483107443	0.753690746	FALSE	0.701313416	Cluster higher
IMD_decile_2020_4_to_7	6	0.340073278	0.389752646	-0.049679368	-0.111622993	0.012264256	0.12494679	0.37484037	FALSE	-1.534336909	Cluster lower
IMD_decile_2020_8_to_10	1	0.331415312	0.304945311	0.026470001	-0.014701715	0.067641716	0.201727701	0.502418452	FALSE	1.276644735	Cluster higher
IMD_decile_2020_8_to_10	2	0.304305265	0.304945311	-6.40E-04	-0.037018307	0.035738215	0.972499429	0.986616738	FALSE	-0.034473681	Cluster lower
IMD_decile_2020_8_to_10	3	0.278306825	0.304945311	-0.026638486	-0.077370538	0.024093566	0.313351887	0.682433656	FALSE	-1.00821369	Cluster lower

IMD_decile_2020_8_to_10	4	0.300576078	0.304945311	-0.004369233	-0.058162484	0.049424019	0.873913274	0.934883967	FALSE	-0.158689801	Cluster lower
IMD_decile_2020_8_to_10	5	0.285343638	0.304945311	-0.019601673	-0.096585267	0.057381921	0.623787703	0.797062065	FALSE	-0.490489295	Cluster lower
IMD_decile_2020_8_to_10	6	0.294140832	0.304945311	-0.010804479	-0.070279342	0.048670384	0.724048765	0.855346499	FALSE	-0.353052924	Cluster lower
NET_Midlands	1	0.124071716	0.131533882	-0.007462166	-0.036570258	0.021645927	0.620609319	0.797062065	FALSE	-0.494986968	Cluster lower
NET_Midlands	2	0.141187233	0.131533882	0.009653351	-0.017676019	0.036982721	0.482203855	0.753690746	FALSE	0.702762365	Cluster higher
NET_Midlands	3	0.144050904	0.131533882	0.012517022	-0.026922011	0.051956055	0.522011786	0.76631616	FALSE	0.640247378	Cluster higher
NET_Midlands	4	0.117214087	0.131533882	-0.014319795	-0.052261804	0.023622214	0.476565934	0.753690746	FALSE	-0.711836651	Cluster lower
NET_Midlands	5	0.142400475	0.131533882	0.010866593	-0.048543378	0.070276563	0.711873916	0.855346499	FALSE	0.369340534	Cluster higher
NET_Midlands	6	0.113213467	0.131533882	-0.018320416	-0.059896822	0.023255991	0.412711976	0.753690746	FALSE	-0.819130475	Cluster lower
NET_North	1	0.249468533	0.259619974	-0.010151441	-0.048264904	0.027962023	0.604516961	0.797062065	FALSE	-0.517915881	Cluster lower
NET_North	2	0.247852833	0.259619974	-0.01176714	-0.046030177	0.022495896	0.50420465	0.764618041	FALSE	-0.667888686	Cluster lower
NET_North	3	0.261547068	0.259619974	0.001927094	-0.047640996	0.051495185	0.93915089	0.968873097	FALSE	0.076337126	Cluster higher
NET_North	4	0.265088292	0.259619974	0.005468318	-0.046252743	0.057189379	0.834993697	0.923413043	FALSE	0.208301325	Cluster higher
NET_North	5	0.248409477	0.259619974	-0.011210497	-0.084852654	0.06243166	0.768372691	0.898277669	FALSE	-0.294504153	Cluster lower
NET_North	6	0.320443307	0.259619974	0.060823333	2.97E-04	0.121349987	0.038354869	0.155675645	FALSE	2.071042024	Cluster higher
NET_South	1	0.221950344	0.208649805	0.013300539	-0.023044726	0.049645804	0.467292113	0.753690746	FALSE	0.726892009	Cluster higher
NET_South	2	0.225184054	0.208649805	0.016534249	-0.016266372	0.04933487	0.316490971	0.682433656	FALSE	1.001694899	Cluster higher
NET_South	3	0.186291149	0.208649805	-0.022358656	-0.066522077	0.021804766	0.336791037	0.704199442	FALSE	-0.960525035	Cluster lower
NET_South	4	0.203669373	0.208649805	-0.004980432	-0.052255252	0.042294388	0.837528865	0.923413043	FALSE	-0.20505537	Cluster lower
NET_South	5	0.20166691	0.208649805	-0.006982895	-0.075360732	0.061394942	0.843116256	0.923413043	FALSE	-0.197909008	Cluster lower
NET_South	6	0.162141141	0.208649805	-0.046508664	-0.095040582	0.002023253	0.083030443	0.279468321	FALSE	-1.73336709	Cluster lower
Northern_Ireland	1	0.03768336	0.029384476	0.008298883	-0.008068418	0.024666185	0.28573372	0.646413989	FALSE	1.067527461	Cluster higher
Northern_Ireland	2	0.022440552	0.029384476	-0.006943924	-0.019079208	0.005191359	0.29387503	0.654108939	FALSE	-1.049658761	Cluster lower
Northern_Ireland	3	0.030107596	0.029384476	7.23E-04	-0.018528921	0.01997516	0.940789819	0.968873097	FALSE	0.0742772	Cluster higher
Northern_Ireland	4	0.046073869	0.029384476	0.016689393	-0.00740546	0.040784246	0.108953842	0.352530788	FALSE	1.602913094	Cluster higher
Northern_Ireland	5	0.017883419	0.029384476	-0.011501058	-0.034442389	0.011440274	0.428458812	0.753690746	FALSE	-0.791831706	Cluster lower
Northern_Ireland	6	0.01575788	0.029384476	-0.013626596	-0.030474296	0.003221103	0.215589145	0.512953483	FALSE	-1.238342356	Cluster lower
Scotland	1	0.082052681	0.090252627	-0.008199946	-0.032525934	0.016126042	0.519722754	0.76631616	FALSE	-0.643772831	Cluster lower
Scotland	2	0.079797424	0.090252627	-0.010455203	-0.032187489	0.011277083	0.359361841	0.740178119	FALSE	-0.91658177	Cluster lower
Scotland	3	0.090645642	0.090252627	3.93E-04	-0.031990866	0.032776897	0.980994865	0.986616738	FALSE	0.023821657	Cluster higher

Scotland	4	0.093373395	0.090252627	0.003120768	-0.030941261	0.037182797	0.8558098	0.92655914	FALSE	0.18171066	Cluster higher
Scotland	5	0.134865746	0.090252627	0.044613118	-0.013096343	0.10232258	0.07655126	0.270873689	FALSE	1.771056781	Cluster higher
Scotland	6	0.115537994	0.090252627	0.025285366	-0.016026437	0.06659717	0.189726852	0.494005765	FALSE	1.311387583	Cluster higher
Wales	1	0.044909894	0.049982834	-0.00507294	-0.023460778	0.013314898	0.599946571	0.797062065	FALSE	-0.524477347	Cluster lower
Wales	2	0.046030577	0.049982834	-0.003952257	-0.020683594	0.012779081	0.649895203	0.810122223	FALSE	-0.453907781	Cluster lower
Wales	3	0.04559564	0.049982834	-0.004387194	-0.028052408	0.019278019	0.725185075	0.855346499	FALSE	-0.351537592	Cluster lower
Wales	4	0.062929956	0.049982834	0.012947121	-0.015216565	0.041110808	0.327263617	0.694805833	FALSE	0.979640478	Cluster higher
Wales	5	0.065660383	0.049982834	0.015677549	-0.026248447	0.057603544	0.4113358	0.753690746	FALSE	0.821545181	Cluster higher
Wales	6	0.056518779	0.049982834	0.006535945	-0.023426438	0.036498327	0.653939261	0.810122223	FALSE	0.448296451	Cluster higher
**age_cross_18.34**	**1**	**0.276745073**	**0.355525191**	**-0.078780118**	**-0.118647868**	**-0.038912369**	**2.06E-04**	**0.002848094**	**TRUE**	**-3.711071699**	**Cluster lower**
age_cross_18.34	2	0.37129077	0.355525191	0.015765579	-0.022341298	0.053872456	0.415290207	0.753690746	FALSE	0.814619391	Cluster higher
**age_cross_18.34**	**3**	**0.46820194**	**0.355525191**	**0.112676749**	**0.056653476**	**0.168700022**	**4.99E-05**	**8.61E-04**	**TRUE**	**4.056135866**	**Cluster higher**
**age_cross_18.34**	**4**	**0.532889646**	**0.355525191**	**0.177364455**	**0.119119302**	**0.235609607**	**9.26E-10**	**1.28E-07**	**TRUE**	**6.121712733**	**Cluster higher**
**age_cross_18.34**	**5**	**0.11788101**	**0.355525191**	**-0.237644181**	**-0.294293285**	**-0.180995077**	**8.31E-09**	**5.73E-07**	**TRUE**	**-5.7620523**	**Cluster lower**
**age_cross_18.34**	**6**	**0.254456961**	**0.355525191**	**-0.10106823**	**-0.158399463**	**-0.043736997**	**0.001409781**	**0.009264277**	**TRUE**	**-3.19264093**	**Cluster lower**
age_cross_35.54	1	0.386609698	0.377144617	0.00946508	-0.033274096	0.052204257	0.663360951	0.810122223	FALSE	0.435277588	Cluster higher
age_cross_35.54	2	0.363359208	0.377144617	-0.01378541	-0.051879583	0.024308764	0.479897362	0.753690746	FALSE	-0.706467652	Cluster lower
age_cross_35.54	3	0.39358071	0.377144617	0.016436093	-0.038629281	0.071501467	0.556186109	0.783200847	FALSE	0.588515835	Cluster higher
age_cross_35.54	4	0.345878699	0.377144617	-0.031265919	-0.087164847	0.02463301	0.280055159	0.644126865	FALSE	-1.08019544	Cluster lower
age_cross_35.54	5	0.455618422	0.377144617	0.078473805	-0.006219571	0.163167181	0.062964068	0.228658985	FALSE	1.859445142	Cluster higher
age_cross_35.54	6	0.371434765	0.377144617	-0.005709852	-0.068736352	0.057316647	0.859417173	0.92655914	FALSE	-0.177116129	Cluster lower
**age_cross_55_or_above**	**1**	**0.33664523**	**0.267330192**	**0.069315038**	**0.028315522**	**0.110314554**	**5.82E-04**	**0.005576122**	**TRUE**	**3.439750775**	**Cluster higher**
age_cross_55_or_above	2	0.265350022	0.267330192	-0.001980169	-0.036903767	0.032943428	0.911618794	0.954476848	FALSE	-0.110996914	Cluster lower
**age_cross_55_or_above**	**3**	**0.13821735**	**0.267330192**	**-0.129112842**	**-0.169545134**	**-0.08868055**	**2.36E-07**	**5.42E-06**	**TRUE**	**-5.16859401**	**Cluster lower**
**age_cross_55_or_above**	**4**	**0.121231656**	**0.267330192**	**-0.146098536**	**-0.186137257**	**-0.106059815**	**1.78E-08**	**8.18E-07**	**TRUE**	**-5.632311071**	**Cluster lower**
**age_cross_55_or_above**	**5**	**0.426500567**	**0.267330192**	**0.159170376**	**0.075394542**	**0.242946209**	**4.15E-05**	**8.18E-04**	**TRUE**	**4.098904976**	**Cluster higher**
**age_cross_55_or_above**	**6**	**0.374108274**	**0.267330192**	**0.106778082**	**0.044148056**	**0.169408109**	**3.36E-04**	**0.004211447**	**TRUE**	**3.586072957**	**Cluster higher**
ethnicity_Asian	1	0.010083099	0.022511565	-0.012428466	-0.022206533	-0.002650398	0.047755064	0.186609693	FALSE	-1.979541572	Cluster lower
ethnicity_Asian	2	0.020863809	0.022511565	-0.001647756	-0.013053858	0.009758347	0.781111016	0.898277669	FALSE	-0.277871482	Cluster lower
ethnicity_Asian	3	0.045217143	0.022511565	0.022705578	-2.80E-05	0.045439183	0.011958053	0.063469668	FALSE	2.513379757	Cluster higher
ethnicity_Asian	4	0.030445344	0.022511565	0.007933779	-0.011923734	0.027791292	0.380428142	0.753690746	FALSE	0.877107701	Cluster higher
ethnicity_Asian	5	0.012871816	0.022511565	-0.009639748	-0.029206744	0.009927247	0.44941806	0.753690746	FALSE	-0.756385565	Cluster lower

ethnicity_Asian	6	0.023050312	0.022511565	5.39E-04	-0.019009545	0.02008704	0.956513631	0.97776949	FALSE	0.054529092	Cluster higher
ethnicity_Black	1	0.004967374	0.016881475	-0.011914102	-0.019407714	-0.00442049	0.026150031	0.120290143	FALSE	-2.223976414	Cluster lower
ethnicity_Black	2	0.024131665	0.016881475	0.00725019	-0.004429743	0.018930123	0.184223819	0.488901674	FALSE	1.32786164	Cluster higher
ethnicity_Black	3	0.033781879	0.016881475	0.016900404	-0.002865166	0.036665974	0.031223048	0.138992921	FALSE	2.154218413	Cluster higher
ethnicity_Black	4	0.01142437	0.016881475	-0.005457105	-0.018244055	0.007329845	0.470923215	0.753690746	FALSE	-0.720977733	Cluster lower
ethnicity_Black	5	0.011183465	0.016881475	-0.00569801	-0.023843774	0.012447755	0.607529964	0.797062065	FALSE	-0.513602454	Cluster lower
ethnicity_Black	6	0.009653292	0.016881475	-0.007228183	-0.020397908	0.005941542	0.389833954	0.753690746	FALSE	-0.859918529	Cluster lower
ethnicity_Mixed	1	0.021123798	0.026120963	-0.004997164	-0.017917393	0.007923065	0.47618812	0.753690746	FALSE	-0.712446839	Cluster lower
ethnicity_Mixed	2	0.026013108	0.026120963	-1.08E-04	-0.012697215	0.012481505	0.986616738	0.986616738	FALSE	-0.016774218	Cluster lower
ethnicity_Mixed	3	0.031364473	0.026120963	0.00524351	-0.014226195	0.024713215	0.572431254	0.788195419	FALSE	0.564474494	Cluster higher
ethnicity_Mixed	4	0.043453683	0.026120963	0.01733272	-0.00604844	0.040713881	0.079447737	0.274094694	FALSE	1.753899453	Cluster higher
ethnicity_Mixed	5	0.009155733	0.026120963	-0.01696523	-0.033947353	1.69E-05	0.212996627	0.512953483	FALSE	-1.245367684	Cluster lower
ethnicity_Mixed	6	0.0193446	0.026120963	-0.006776363	-0.025013231	0.011460505	0.518256805	0.76631616	FALSE	-0.646034822	Cluster lower
**ethnicity_NET_ethnic_minority**	**1**	**0.038638234**	**0.067661011**	**-0.029022777**	**-0.04709887**	**-0.010946684**	**0.007185044**	**0.041314005**	**TRUE**	**-2.68814382**	**Cluster lower**
ethnicity_NET_ethnic_minority	2	0.072505282	0.067661011	0.004844271	-0.015498172	0.025186714	0.635269686	0.804286391	FALSE	0.47432287	Cluster higher
**ethnicity_NET_ethnic_minority**	**3**	**0.114126578**	**0.067661011**	**0.046465567**	**0.011420781**	**0.081510353**	**0.001954062**	**0.012257297**	**TRUE**	**3.097126936**	**Cluster higher**
ethnicity_NET_ethnic_minority	4	0.085323397	0.067661011	0.017662386	-0.014743152	0.050067925	0.246171506	0.57579098	FALSE	1.159698684	Cluster higher
ethnicity_NET_ethnic_minority	5	0.033211014	0.067661011	-0.034449996	-0.065739786	-0.003160207	0.10984655	0.352530788	FALSE	-1.598883234	Cluster lower
ethnicity_NET_ethnic_minority	6	0.057232619	0.067661011	-0.010428392	-0.04095102	0.020094237	0.529902825	0.76631616	FALSE	-0.628154361	Cluster lower
**ethnicity_White**	**1**	**0.945231547**	**0.903940598**	**0.041290949**	**0.019990674**	**0.062591223**	**0.001132463**	**0.008225258**	**TRUE**	**3.255366025**	**Cluster higher**
ethnicity_White	2	0.896566811	0.903940598	-0.007373787	-0.03125687	0.016509296	0.5384011	0.76631616	FALSE	-0.615232632	Cluster lower
**ethnicity_White**	**3**	**0.846004811**	**0.903940598**	**-0.057935788**	**-0.097825077**	**-0.018046498**	**9.33E-04**	**0.007186734**	**TRUE**	**-3.309892009**	**Cluster lower**
ethnicity_White	4	0.875832947	0.903940598	-0.028107651	-0.066338983	0.010123681	0.116089617	0.356008159	FALSE	-1.571400642	Cluster lower
ethnicity_White	5	0.942514134	0.903940598	0.038573536	-0.001698	0.078845071	0.128205347	0.376432722	FALSE	1.521217173	Cluster higher
ethnicity_White	6	0.918715885	0.903940598	0.014775287	-0.021122488	0.050673062	0.44821683	0.753690746	FALSE	0.758391191	Cluster higher
parent_non_parent	1	0.462961795	0.510812713	-0.047850919	-0.091676359	-0.004025478	0.032744928	0.141212502	FALSE	-2.135196924	Cluster lower
parent_non_parent	2	0.494536837	0.510812713	-0.016275877	-0.055797956	0.023246203	0.419586666	0.753690746	FALSE	-0.807138547	Cluster lower
parent_non_parent	3	0.58480017	0.510812713	0.073987456	0.018295697	0.129679216	0.010091583	0.055705539	FALSE	2.57267531	Cluster higher
**parent_non_parent**	**4**	**0.668251771**	**0.510812713**	**0.157439058**	**0.101831219**	**0.213046896**	**1.35E-07**	**3.74E-06**	**TRUE**	**5.271292748**	**Cluster higher**
**parent_non_parent**	**5**	**0.361850557**	**0.510812713**	**-0.148962156**	**-0.230975494**	**-0.066948819**	**6.06E-04**	**0.005576122**	**TRUE**	**-3.428869804**	**Cluster lower**
parent_non_parent	6	0.466362973	0.510812713	-0.04444974	-0.109515254	0.020615774	0.181453475	0.488901674	FALSE	-1.33629315	Cluster lower
**parent_to_child_over_18**	**1**	**0.319456525**	**0.255793745**	**0.06366278**	**0.023215586**	**0.104109973**	**0.001351567**	**0.009264277**	**TRUE**	**3.204799282**	**Cluster higher**

parent_to_child_over_18	2	0.262222361	0.255793745	0.006428616	-0.028273044	0.041130275	0.715433951	0.855346499	FALSE	0.364567938	Cluster higher
**parent_to_child_over_18**	**3**	**0.122488756**	**0.255793745**	**-0.133304989**	**-0.171986103**	**-0.094623875**	**5.78E-08**	**1.99E-06**	**TRUE**	**-5.425412078**	**Cluster lower**
**parent_to_child_over_18**	**4**	**0.156040496**	**0.255793745**	**-0.099753249**	**-0.143361477**	**-0.05614502**	**1.05E-04**	**0.001610144**	**TRUE**	**-3.878715755**	**Cluster lower**
**parent_to_child_over_18**	**5**	**0.371119749**	**0.255793745**	**0.115326004**	**0.033459272**	**0.197192736**	**0.002538686**	**0.015232115**	**TRUE**	**3.01869187**	**Cluster higher**
parent_to_child_over_18	6	0.320879851	0.255793745	0.065086106	0.004563127	0.125609086	0.026047588	0.120290143	FALSE	2.225501531	Cluster higher
parent_to_child_u18	1	0.246680908	0.237089206	0.009591702	-0.028176579	0.047359983	0.615821341	0.797062065	FALSE	0.50178134	Cluster higher
parent_to_child_u18	2	0.23313817	0.237089206	-0.003951036	-0.037425024	0.029522952	0.81757958	0.917284406	FALSE	-0.230659191	Cluster lower
parent_to_child_u18	3	0.285468319	0.237089206	0.048379113	-0.002223778	0.098982003	0.050033034	0.186609693	FALSE	1.959681458	Cluster higher
parent_to_child_u18	4	0.19705162	0.237089206	-0.040037586	-0.087057367	0.006982195	0.113217528	0.355091337	FALSE	-1.583895386	Cluster lower
parent_to_child_u18	5	0.257740853	0.237089206	0.020651647	-0.053726902	0.095030196	0.576867662	0.788195419	FALSE	0.557965912	Cluster higher
parent_to_child_u18	6	0.198250685	0.237089206	-0.038838521	-0.091152532	0.01347549	0.167676382	0.462786814	FALSE	-1.379708606	Cluster lower
social_grade_ABC1	1	0.633437664	0.579732665	0.053704999	0.011165623	0.096244375	0.01482426	0.073062423	FALSE	2.436644144	Cluster higher
social_grade_ABC1	2	0.604974412	0.579732665	0.025241747	-0.013496562	0.063980056	0.203879951	0.502418452	FALSE	1.270574815	Cluster higher
social_grade_ABC1	3	0.582837369	0.579732665	0.003104704	-0.052533482	0.058742891	0.912977854	0.954476848	FALSE	0.109283221	Cluster higher
social_grade_ABC1	4	0.559238838	0.579732665	-0.020493827	-0.078677154	0.0376895	0.488050667	0.753690746	FALSE	-0.693412585	Cluster lower
**social_grade_ABC1**	**5**	**0.437494545**	**0.579732665**	**-0.14223812**	**-0.226696873**	**-0.057779367**	**9.37E-04**	**0.007186734**	**TRUE**	**-3.308670745**	**Cluster lower**
**social_grade_ABC1**	**6**	**0.464004985**	**0.579732665**	**-0.11572768**	**-0.180699511**	**-0.05075585**	**4.44E-04**	**0.004710125**	**TRUE**	**-3.51262247**	**Cluster lower**
social_grade_C2DE	1	0.366562336	0.420267335	-0.053704999	-0.096244375	-0.011165623	0.01482426	0.073062423	FALSE	-2.436644144	Cluster lower
social_grade_C2DE	2	0.395025588	0.420267335	-0.025241747	-0.063980056	0.013496562	0.203879951	0.502418452	FALSE	-1.270574815	Cluster lower
social_grade_C2DE	3	0.417162631	0.420267335	-0.003104704	-0.058742891	0.052533482	0.912977854	0.954476848	FALSE	-0.109283221	Cluster lower
social_grade_C2DE	4	0.440761162	0.420267335	0.020493827	-0.0376895	0.078677154	0.488050667	0.753690746	FALSE	0.693412585	Cluster higher
**social_grade_C2DE**	**5**	**0.562505455**	**0.420267335**	**0.14223812**	**0.057779367**	**0.226696873**	**9.37E-04**	**0.007186734**	**TRUE**	**3.308670745**	**Cluster higher**
**social_grade_C2DE**	**6**	**0.535995015**	**0.420267335**	**0.11572768**	**0.05075585**	**0.180699511**	**4.44E-04**	**0.004710125**	**TRUE**	**3.51262247**	**Cluster higher**
**A1**	**1**	**0.427548974**	**0.34014084**	**0.087408133**	**0.044310264**	**0.130506003**	**4.73E-05**	**4.26E-04**	**TRUE**	**4.068690003**	**Cluster higher**
A1	2	0.356444852	0.34014084	0.016304012	-0.021456887	0.054064911	0.394861192	0.47383343	FALSE	0.850834684	Cluster higher
**A1**	**3**	**0.27972757**	**0.34014084**	**-0.06041327**	**-0.111404131**	**-0.00942241**	**0.025931249**	**0.049174939**	**TRUE**	**-2.227239839**	**Cluster lower**
**A1**	**4**	**0.13594531**	**0.34014084**	**-0.20419553**	**-0.24639363**	**-0.16199743**	**2.24E-13**	**4.04E-12**	**TRUE**	**-7.333360293**	**Cluster lower**
**A1**	**5**	**0.467570004**	**0.34014084**	**0.127429164**	**0.042682449**	**0.212175879**	**0.002062386**	**0.006187159**	**TRUE**	**3.081098174**	**Cluster higher**
A1	6	0.337991032	0.34014084	-0.002149808	-0.063842109	0.059542492	0.945615986	0.945615986	FALSE	-0.068213117	Cluster lower
**Q6B**	**1**	**0.741536343**	**0.672525009**	**0.069011335**	**0.029982458**	**0.108040212**	**9.05E-04**	**0.003826157**	**TRUE**	**3.31848153**	**Cluster higher**
**Q6B**	**2**	**0.605778831**	**0.672525009**	**-0.066746177**	**-0.105004305**	**-0.028488049**	**4.95E-04**	**0.002967748**	**TRUE**	**-3.483650609**	**Cluster lower**
Q6B	3	0.661774752	0.672525009	-0.010750256	-0.064074688	0.042574175	0.690963208	0.731608103	FALSE	-0.397548257	Cluster lower

**Q6B**	**4**	**0.739934372**	**0.672525009**	**0.067409364**	**0.015551334**	**0.119267394**	**0.015716608**	**0.035362368**	**TRUE**	**2.415430813**	**Cluster higher**
Q6B	5	0.746224212	0.672525009	0.073699203	-7.31E-04	0.148129127	0.069822096	0.096676749	FALSE	1.813063056	Cluster higher
Q6B	6	0.607018375	0.672525009	-0.065506634	-0.128974255	-0.002039012	0.036757616	0.060148826	FALSE	-2.088446068	Cluster lower
**Q6C**	**1**	**0.428389315**	**0.373190378**	**0.055198937**	**0.011922901**	**0.098474973**	**0.011346034**	**0.029175517**	**TRUE**	**2.531856722**	**Cluster higher**
**Q6C**	**2**	**0.330397106**	**0.373190378**	**-0.042793272**	**-0.08023867**	**-0.005347875**	**0.027319411**	**0.049174939**	**TRUE**	**-2.206923409**	**Cluster lower**
Q6C	3	0.393212849	0.373190378	0.020022471	-0.035021081	0.075066022	0.472604056	0.531679563	FALSE	0.718248535	Cluster higher
Q6C	4	0.319469606	0.373190378	-0.053720772	-0.108632393	0.00119085	0.062508061	0.093762092	FALSE	-1.862674604	Cluster lower
**Q6C**	**5**	**0.511280576**	**0.373190378**	**0.138090198**	**0.053107674**	**0.223072722**	**0.001062821**	**0.003826157**	**TRUE**	**3.273346431**	**Cluster higher**
Q6C	6	0.338174758	0.373190378	-0.03501562	-0.096833915	0.026802674	0.275685614	0.354452932	FALSE	-1.0900625	Cluster lower
**T1**	**1**	**0.142899205**	**0.186668419**	**-0.043769214**	**-0.075242262**	**-0.012296167**	**0.010665401**	**0.032075841**	**TRUE**	**-2.553474767**	**Cluster lower**
T1	2	0.205520225	0.186668419	0.018851805	-0.01281335	0.050516961	0.234733599	0.352100399	FALSE	1.188253379	Cluster higher
**T1**	**3**	**0.24269561**	**0.186668419**	**0.056027191**	**0.008172688**	**0.103881693**	**0.013740354**	**0.037878814**	**TRUE**	**2.463980622**	**Cluster higher**
T1	4	0.172708318	0.186668419	-0.013960102	-0.058442523	0.030522319	0.547999573	0.66025074	FALSE	-0.600760416	Cluster lower
T1	5	0.215474894	0.186668419	0.028806474	-0.041021573	0.098634522	0.396334957	0.506416525	FALSE	0.848184988	Cluster higher
T1	6	0.158355887	0.186668419	-0.028312532	-0.076219136	0.019594072	0.272206744	0.396644112	FALSE	-1.097994837	Cluster lower
**T2**	**1**	**0.039986251**	**0.08445727**	**-0.044471019**	**-0.063350097**	**-0.025591941**	**1.74E-04**	**9.33E-04**	**TRUE**	**-3.754309101**	**Cluster lower**
T2	2	0.102677446	0.08445727	0.018220176	-0.00529335	0.041733702	0.112565257	0.205029575	FALSE	1.586767744	Cluster higher
**T2**	**3**	**0.131817051**	**0.08445727**	**0.04735978**	**0.009952211**	**0.084767349**	**0.004079744**	**0.01600515**	**TRUE**	**2.871929154**	**Cluster higher**
T2	4	0.086029385	0.08445727	0.001572114	-0.031280111	0.034424339	0.924789665	0.933945998	FALSE	0.094402204	Cluster higher
T2	5	0.074247455	0.08445727	-0.010209815	-0.055005861	0.034586232	0.671703632	0.725868916	FALSE	-0.423811032	Cluster lower
T2	6	0.074256982	0.08445727	-0.010200288	-0.0445837	0.024183123	0.579505158	0.676501251	FALSE	-0.554107675	Cluster lower
**T3**	**1**	**0.015328277**	**0.053148505**	**-0.037820227**	**-0.050894066**	**-0.024746389**	**5.21E-05**	**3.32E-04**	**TRUE**	**-4.045826295**	**Cluster lower**
T3	2	0.067235832	0.053148505	0.014087328	-0.005220017	0.033394673	0.130765977	0.230263846	FALSE	1.511088213	Cluster higher
**T3**	**3**	**0.123020655**	**0.053148505**	**0.06987215**	**0.034035082**	**0.105709219**	**4.48E-07**	**5.07E-06**	**TRUE**	**5.047437931**	**Cluster higher**
T3	4	0.047115445	0.053148505	-0.00603306	-0.031048766	0.018982647	0.65110934	0.721882095	FALSE	-0.452221612	Cluster lower
T3	5	0.012340204	0.053148505	-0.040808301	-0.061116038	-0.020500563	0.032730733	0.070045888	FALSE	-2.135370814	Cluster lower
T3	6	0.035612262	0.053148505	-0.017536243	-0.04220825	0.007135764	0.233280194	0.352100399	FALSE	-1.191951676	Cluster lower
**T4**	**1**	**0.127874811**	**0.186248856**	**-0.058374045**	**-0.088747164**	**-0.028000925**	**6.01E-04**	**0.002788032**	**TRUE**	**-3.431009205**	**Cluster lower**
T4	2	0.206589773	0.186248856	0.020340917	-0.011364495	0.052046328	0.199756636	0.328631885	FALSE	1.282245226	Cluster higher
T4	3	0.237889592	0.186248856	0.051640736	0.004083089	0.099198384	0.022885648	0.056280373	FALSE	2.275338224	Cluster higher
**T4**	**4**	**0.246525835**	**0.186248856**	**0.060276979**	**0.010261217**	**0.110292741**	**0.010691947**	**0.032075841**	**TRUE**	**2.552608935**	**Cluster higher**
T4	5	0.173432488	0.186248856	-0.012816368	-0.077393095	0.051760358	0.70437239	0.740680245	FALSE	-0.379424864	Cluster lower

T4	6	0.125103686	0.186248856	-0.061145171	-0.105034729	-0.017255613	0.016915623	0.043134839	TRUE	-2.388536607	Cluster lower
T5	1	0.08231089	0.123930232	-0.041619342	-0.066798387	-0.016440298	0.003682814	0.015025879	TRUE	-2.904124777	Cluster lower
T5	2	0.129626249	0.123930232	0.005696017	-0.020732975	0.032125009	0.669726993	0.725868916	FALSE	0.426522724	Cluster higher
T5	3	0.205645099	0.123930232	0.081714867	0.037053549	0.126376185	3.06E-05	2.21E-04	TRUE	4.169169495	Cluster higher
T5	4	0.154053455	0.123930232	0.030123223	-0.011811737	0.072058183	0.130934344	0.230263846	FALSE	1.510427626	Cluster higher
T5	5	0.094477571	0.123930232	-0.029452662	-0.079619375	0.020714052	0.300954987	0.426352898	FALSE	-1.034387625	Cluster lower
T5	6	0.074931928	0.123930232	-0.048998304	-0.084203233	-0.013793376	0.023174271	0.056280373	FALSE	-2.270549371	Cluster lower
T6	1	0.03307144	0.045187125	-0.012115686	-0.028364968	0.004133597	0.181524835	0.313822596	FALSE	-1.336074772	Cluster lower
T6	2	0.024706252	0.045187125	-0.020480873	-0.033898056	-0.00706369	0.009667326	0.030814601	TRUE	-2.58750691	Cluster lower
T6	3	0.040222857	0.045187125	-0.004964268	-0.027289309	0.017360773	0.676054382	0.725868916	FALSE	-0.417853295	Cluster lower
T6	4	0.111639784	0.045187125	0.066452659	0.030713815	0.102191503	6.60E-07	6.73E-06	TRUE	4.97285686	Cluster higher
T6	5	0.011145735	0.045187125	-0.034041391	-0.053258184	-0.014824597	0.054433239	0.104758309	FALSE	-1.923372868	Cluster lower
T6	6	0.08471375	0.045187125	0.039526625	0.003954807	0.075098443	0.005815693	0.021185737	TRUE	2.757995913	Cluster higher
T7	1	0.063168888	0.074806559	-0.01163767	-0.033367846	0.010092505	0.316616754	0.437564771	FALSE	-1.001434577	Cluster lower
T7	2	0.079161855	0.074806559	0.004355296	-0.016854305	0.025564897	0.683443392	0.726158604	FALSE	0.407768841	Cluster higher
T7	3	0.087750905	0.074806559	0.012944346	-0.018713001	0.044601694	0.397189431	0.506416525	FALSE	0.846651443	Cluster higher
T7	4	0.059240943	0.074806559	-0.015565615	-0.043623428	0.012492198	0.317448952	0.437564771	FALSE	-0.999713965	Cluster lower
T7	5	0.104964351	0.074806559	0.030157793	-0.021672419	0.081988005	0.191378588	0.320010098	FALSE	1.306511743	Cluster higher
T7	6	0.074482355	0.074806559	-3.24E-04	-0.034568119	0.033919712	0.985219531	0.985219531	FALSE	-0.01852563	Cluster lower
MH_5_1	1	0.373712258	0.523339269	-0.149627011	-0.192406008	-0.106848013	2.44E-11	2.48E-09	TRUE	-6.67720116	Cluster lower
MH_5_1	2	0.544956097	0.523339269	0.021616828	-0.017778188	0.061011844	0.282969826	0.406520032	FALSE	1.073671753	Cluster higher
MH_5_1	3	0.603316934	0.523339269	0.079977665	0.024642721	0.13531261	0.005354829	0.020229353	TRUE	2.784875453	Cluster higher
MH_5_1	4	0.682693662	0.523339269	0.159354394	0.104311641	0.214397147	9.04E-08	1.15E-06	TRUE	5.344961542	Cluster higher
MH_5_1	5	0.629074739	0.523339269	0.10573547	0.023321231	0.188149709	0.014772157	0.039651579	TRUE	2.43791721	Cluster higher
MH_5_1	6	0.455168616	0.523339269	-0.068170652	-0.133126641	-0.003214664	0.040322211	0.079093567	FALSE	-2.050432846	Cluster lower
MH_5_2	1	0.432347931	0.552417187	-0.120069257	-0.16362179	-0.076516723	8.03E-08	1.15E-06	TRUE	-5.366344002	Cluster lower
MH_5_2	2	0.558257375	0.552417187	0.005840187	-0.03342946	0.045109835	0.770829828	0.794188308	FALSE	0.291289616	Cluster higher
MH_5_2	3	0.656355621	0.552417187	0.103938434	0.050044759	0.157832108	2.70E-04	0.001312802	TRUE	3.642233386	Cluster higher
MH_5_2	4	0.726719955	0.552417187	0.174302768	0.121328433	0.227277103	3.91E-09	9.98E-08	TRUE	5.887796318	Cluster higher
MH_5_2	5	0.498622672	0.552417187	-0.053794516	-0.138918404	0.031329373	0.213363958	0.344451342	FALSE	-1.244368535	Cluster lower
MH_5_2	6	0.499830248	0.552417187	-0.052586939	-0.117755541	0.012581663	0.112286159	0.205029575	FALSE	-1.588000797	Cluster lower
MH_5_3	1	0.400517263	0.538813788	-0.138296525	-0.181492669	-0.095100381	6.72E-10	3.18E-08	TRUE	-6.172555764	Cluster lower

MH_5_3	2	0.546231701	0.538813788	0.007417912	-0.031951286	0.046787111	0.712090405	0.741155319	FALSE	0.36905007	Cluster higher
**MH_5_3**	**3**	**0.624928913**	**0.538813788**	**0.086115124**	**0.031294468**	**0.140935781**	**0.002641343**	**0.011225706**	**TRUE**	**3.006660851**	**Cluster higher**
**MH_5_3**	**4**	**0.711855329**	**0.538813788**	**0.173041541**	**0.11930352**	**0.226779562**	**5.75E-09**	**1.17E-07**	**TRUE**	**5.823934575**	**Cluster higher**
MH_5_3	5	0.631105152	0.538813788	0.092291363	0.009974195	0.174608531	0.032962771	0.070045888	FALSE	2.132536455	Cluster higher
MH_5_3	6	0.470678527	0.538813788	-0.068135261	-0.133216548	-0.003053974	0.040117555	0.079093567	FALSE	-2.05253647	Cluster lower
**MH_5_4**	**1**	**0.419072487**	**0.5559979**	**-0.136925413**	**-0.180331052**	**-0.093519774**	**9.37E-10**	**3.18E-08**	**TRUE**	**-6.119852142**	**Cluster lower**
MH_5_4	2	0.580748806	0.5559979	0.024750907	-0.014323368	0.063825182	0.216126332	0.344451342	FALSE	1.236894285	Cluster higher
MH_5_4	3	0.618523983	0.5559979	0.062526084	0.007569514	0.117482653	0.028508039	0.064174129	FALSE	2.190223399	Cluster higher
**MH_5_4**	**4**	**0.701093551**	**0.5559979**	**0.145095651**	**0.090878467**	**0.199312835**	**9.50E-07**	**8.81E-06**	**TRUE**	**4.901636381**	**Cluster higher**
MH_5_4	5	0.581204735	0.5559979	0.025206835	-0.058843611	0.109257281	0.559176698	0.663209572	FALSE	0.584064827	Cluster higher
MH_5_4	6	0.533237063	0.5559979	-0.022760837	-0.087793669	0.042271995	0.491319644	0.611153704	FALSE	-0.688211434	Cluster lower
A7	1	0.44295524	0.455246251	-0.012291011	-0.057807042	0.03322502	0.597195186	0.676501251	FALSE	-0.528438263	Cluster lower
A7	2	0.467859604	0.455246251	0.012613353	-0.028693765	0.053920471	0.549144291	0.66025074	FALSE	0.59904288	Cluster higher
**A7**	**3**	**0.53309368**	**0.455246251**	**0.077847429**	**0.0194647**	**0.136230158**	**0.008960379**	**0.029482537**	**TRUE**	**2.613562137**	**Cluster higher**
**A7**	**4**	**0.319579394**	**0.455246251**	**-0.135666857**	**-0.192431714**	**-0.078902001**	**9.14E-06**	**7.77E-05**	**TRUE**	**-4.4366771**	**Cluster lower**
**A7**	**5**	**0.595640122**	**0.455246251**	**0.140393871**	**0.051402289**	**0.229385453**	**0.002320086**	**0.010289076**	**TRUE**	**3.045869416**	**Cluster higher**
A7	6	0.43615959	0.455246251	-0.019086661	-0.088396754	0.050223432	0.590690675	0.676501251	FALSE	-0.537835421	Cluster lower
**DV3_1**	**1**	**0.116160747**	**0.190359328**	**-0.074198582**	**-0.103725387**	**-0.044671776**	**1.35E-05**	**1.06E-04**	**TRUE**	**-4.351885393**	**Cluster lower**
DV3_1	2	0.209693713	0.190359328	0.019334384	-0.012567272	0.051236041	0.226444865	0.349960246	FALSE	1.209567579	Cluster higher
**DV3_1**	**3**	**0.278730099**	**0.190359328**	**0.088370771**	**0.038506312**	**0.138235229**	**1.28E-04**	**7.51E-04**	**TRUE**	**3.8302029**	**Cluster higher**
DV3_1	4	0.239259337	0.190359328	0.048900009	-6.98E-04	0.098497657	0.039455171	0.079093567	FALSE	2.059407994	Cluster higher
DV3_1	5	0.116446719	0.190359328	-0.073912609	-0.129257482	-0.018567736	0.028941274	0.064174129	FALSE	-2.184285522	Cluster lower
DV3_1	6	0.170195043	0.190359328	-0.020164285	-0.069372908	0.029044338	0.43842381	0.552089243	FALSE	-0.774857597	Cluster lower
**DV3_2**	**1**	**0.188212768**	**0.234574557**	**-0.046361789**	**-0.081296984**	**-0.011426594**	**0.013289514**	**0.037878814**	**TRUE**	**-2.475915194**	**Cluster lower**
DV3_2	2	0.249567605	0.234574557	0.014993048	-0.019042696	0.049028792	0.382918955	0.506416525	FALSE	0.87253065	Cluster higher
**DV3_2**	**3**	**0.30085844**	**0.234574557**	**0.066283882**	**0.015002333**	**0.117565432**	**0.007226415**	**0.025417047**	**TRUE**	**2.686226182**	**Cluster higher**
DV3_2	4	0.276685903	0.234574557	0.042111345	-0.010045719	0.094268409	0.099020297	0.187038338	FALSE	1.649621881	Cluster higher
**DV3_2**	**5**	**0.14427401**	**0.234574557**	**-0.090300547**	**-0.150857794**	**-0.0297433**	**0.013494277**	**0.037878814**	**TRUE**	**-2.470451097**	**Cluster lower**
DV3_2	6	0.207309377	0.234574557	-0.02726518	-0.080349535	0.025819175	0.331699587	0.445175761	FALSE	-0.970696196	Cluster lower
**DV3_3**	**1**	**0.063135814**	**0.121328345**	**-0.05819253**	**-0.081210283**	**-0.035174778**	**3.25E-05**	**2.21E-04**	**TRUE**	**-4.154852139**	**Cluster lower**
DV3_3	2	0.151103021	0.121328345	0.029774677	0.002059702	0.057489651	0.027485759	0.063716987	FALSE	2.204548981	Cluster higher
DV3_3	3	0.161603061	0.121328345	0.040274716	-7.12E-04	0.081261872	0.035133696	0.073135449	FALSE	2.106814107	Cluster higher
DV3_3	4	0.131771936	0.121328345	0.010443591	-0.029069277	0.049956459	0.594461808	0.676501251	FALSE	0.532381482	Cluster higher

DV3_3	5	0.128410202	0.121328345	0.007081857	-0.049818636	0.06398235	0.803134409	0.819197097	FALSE	0.249292659	Cluster higher
DV3_3	6	0.096345905	0.121328345	-0.024982439	-0.06385013	0.013885251	0.246280733	0.36406717	FALSE	-1.159430543	Cluster lower
**DV3_4**	**1**	**0.157183857**	**0.225637228**	**-0.068453371**	**-0.101432944**	**-0.035473799**	**1.86E-04**	**9.49E-04**	**TRUE**	**-3.737087727**	**Cluster lower**
DV3_4	2	0.205275503	0.225637228	-0.020361725	-0.05257037	0.01184692	0.223419329	0.349960246	FALSE	-1.217486082	Cluster lower
**DV3_4**	**3**	**0.361336655**	**0.225637228**	**0.135699427**	**0.082306413**	**0.189092442**	**3.73E-08**	**6.34E-07**	**TRUE**	**5.503298072**	**Cluster higher**
**DV3_4**	**4**	**0.322810316**	**0.225637228**	**0.097173087**	**0.042966143**	**0.151380032**	**1.33E-04**	**7.51E-04**	**TRUE**	**3.821705178**	**Cluster higher**
**DV3_4**	**5**	**0.139248399**	**0.225637228**	**-0.086388829**	**-0.146061792**	**-0.026715866**	**0.016570094**	**0.043134839**	**TRUE**	**-2.396110492**	**Cluster lower**
DV3_4	6	0.202006901	0.225637228	-0.023630327	-0.076185757	0.028925103	0.393897786	0.506416525	FALSE	-0.852570034	Cluster lower
T12	1	0.586978359	0.598399354	-0.011420995	-0.054637446	0.031795457	0.603545233	0.676501251	FALSE	-0.519309064	Cluster lower
T12	2	0.64271088	0.598399354	0.044311526	0.006212285	0.082410767	0.024336637	0.057728767	FALSE	2.251772744	Cluster higher
T12	3	0.626286728	0.598399354	0.027887374	-0.026783042	0.082557791	0.322342105	0.438385263	FALSE	0.989656374	Cluster higher
**T12**	**4**	**0.520745792**	**0.598399354**	**-0.077653561**	**-0.136115023**	**-0.0191921**	**0.00832718**	**0.028312411**	**TRUE**	**-2.638507745**	**Cluster lower**
T12	5	0.572937279	0.598399354	-0.025462075	-0.109660347	0.058736198	0.55020895	0.66025074	FALSE	-0.597447048	Cluster lower
T12	6	0.555509127	0.598399354	-0.042890227	-0.107609586	0.021829132	0.189198056	0.320010098	FALSE	-1.312955165	Cluster lower
**A11_1**	**1**	**0.766834742**	**0.564025198**	**0.202809544**	**0.16429277**	**0.241326317**	**2.15E-20**	**5.23E-20**	**TRUE**	**9.254644535**	**Cluster higher**
**A11_1**	**2**	**0.393122846**	**0.564025198**	**-0.170902352**	**-0.209661224**	**-0.13214348**	**2.20E-17**	**4.22E-17**	**TRUE**	**-8.482491546**	**Cluster lower**
**A11_1**	**3**	**0.899068791**	**0.564025198**	**0.335043593**	**0.297696077**	**0.372391109**	**1.12E-32**	**6.28E-32**	**TRUE**	**11.90487663**	**Cluster higher**
**A11_1**	**4**	**0.674684115**	**0.564025198**	**0.110658916**	**0.05534932**	**0.165968513**	**1.82E-04**	**2.21E-04**	**TRUE**	**3.743022722**	**Cluster higher**
**A11_1**	**5**	**0.107407873**	**0.564025198**	**-0.456617326**	**-0.511501518**	**-0.401733134**	**5.43E-26**	**1.81E-25**	**TRUE**	**-10.54370089**	**Cluster lower**
**A11_1**	**6**	**0.271501794**	**0.564025198**	**-0.292523405**	**-0.351155339**	**-0.23389147**	**1.16E-18**	**2.44E-18**	**TRUE**	**-8.818084655**	**Cluster lower**
**A11_10**	**1**	**0.648362003**	**0.521749424**	**0.126612578**	**0.084271871**	**0.168953285**	**1.39E-08**	**1.98E-08**	**TRUE**	**5.674910784**	**Cluster higher**
A11_10	2	0.513128235	0.521749424	-0.008621189	-0.048127823	0.030885444	0.668779082	0.699885086	FALSE	-0.427824248	Cluster lower
**A11_10**	**3**	**0.849308379**	**0.521749424**	**0.327558955**	**0.284933837**	**0.370184072**	**1.91E-30**	**8.59E-30**	**TRUE**	**11.46801199**	**Cluster higher**
**A11_10**	**4**	**0.316975724**	**0.521749424**	**-0.204773701**	**-0.259804052**	**-0.14974335**	**7.65E-12**	**1.30E-11**	**TRUE**	**-6.84499724**	**Cluster lower**
**A11_10**	**5**	**0.321553047**	**0.521749424**	**-0.200196377**	**-0.280042174**	**-0.12035058**	**4.04E-06**	**5.20E-06**	**TRUE**	**-4.609255325**	**Cluster lower**
**A11_10**	**6**	**0.152202389**	**0.521749424**	**-0.369547036**	**-0.418336899**	**-0.320757173**	**1.10E-28**	**4.30E-28**	**TRUE**	**-11.11179188**	**Cluster lower**
**A11_11**	**1**	**0.505576549**	**0.353941884**	**0.151634665**	**0.108089791**	**0.19517954**	**3.53E-12**	**6.10E-12**	**TRUE**	**6.954941078**	**Cluster higher**
A11_11	2	0.374869776	0.353941884	0.020927892	-0.0172257	0.059081484	0.279446853	0.295884903	FALSE	1.081562774	Cluster higher
**A11_11**	**3**	**0.515927494**	**0.353941884**	**0.16198561**	**0.105893126**	**0.218078095**	**5.99E-09**	**9.14E-09**	**TRUE**	**5.817029304**	**Cluster higher**
**A11_11**	**4**	**0.076868485**	**0.353941884**	**-0.277073399**	**-0.311995482**	**-0.242151316**	**3.98E-23**	**1.08E-22**	**TRUE**	**-9.904572764**	**Cluster lower**
**A11_11**	**5**	**0.202384937**	**0.353941884**	**-0.151556947**	**-0.220734199**	**-0.082379695**	**2.45E-04**	**2.94E-04**	**TRUE**	**-3.667131626**	**Cluster lower**
**A11_11**	**6**	**0.11766784**	**0.353941884**	**-0.236274044**	**-0.280527975**	**-0.192020113**	**4.77E-14**	**8.58E-14**	**TRUE**	**-7.538196338**	**Cluster lower**
**A11_12**	**1**	**0.819144076**	**0.604652501**	**0.214491575**	**0.178661825**	**0.250321325**	**1.28E-23**	**3.83E-23**	**TRUE**	**10.01744153**	**Cluster higher**

A11_12	2	0.567994426	0.604652501	-0.036658075	-0.075691946	0.002375796	0.064039782	0.070287566	FALSE	-1.851902796	Cluster lower
A11_12	3	0.85795532	0.604652501	0.253302819	0.211681756	0.294923882	6.10E-20	1.41E-19	TRUE	9.142522601	Cluster higher
A11_12	4	0.352177183	0.604652501	-0.252475318	-0.308638512	-0.196312124	1.35E-17	2.64E-17	TRUE	-8.539457994	Cluster lower
A11_12	5	0.523104682	0.604652501	-0.081547819	-0.166511295	0.003415656	0.055362433	0.061513814	FALSE	-1.916020919	Cluster lower
A11_12	6	0.203385656	0.604652501	-0.401266845	-0.454877975	-0.347655715	4.18E-34	2.68E-33	TRUE	-12.17593019	Cluster lower
A11_13	1	0.9277934	0.760096899	0.167696501	0.141328396	0.194064607	2.33E-20	5.53E-20	TRUE	9.245837851	Cluster higher
A11_13	2	0.761129922	0.760096899	0.001033023	-0.032686674	0.03475272	0.952155807	0.952155807	FALSE	0.059999784	Cluster higher
A11_13	3	0.913124977	0.760096899	0.153028079	0.11883815	0.187218007	1.52E-10	2.44E-10	TRUE	6.403760277	Cluster higher
A11_13	4	0.580827996	0.760096899	-0.179268903	-0.236309802	-0.122228004	8.47E-12	1.41E-11	TRUE	-6.83035762	Cluster lower
A11_13	5	0.771033763	0.760096899	0.010936864	-0.060679629	0.082553358	0.767984538	0.785438732	FALSE	0.295012228	Cluster higher
A11_13	6	0.345996553	0.760096899	-0.414100346	-0.475582004	-0.352618688	6.38E-44	5.22E-43	TRUE	-13.89952093	Cluster lower
A11_14	1	0.79364581	0.602757072	0.190888738	0.153729433	0.228048044	6.81E-19	1.46E-18	TRUE	8.878018952	Cluster higher
A11_14	2	0.434362264	0.602757072	-0.168394808	-0.207455428	-0.129334188	3.99E-17	7.48E-17	TRUE	-8.413193926	Cluster lower
A11_14	3	0.921006926	0.602757072	0.318249855	0.283845545	0.352654164	9.53E-31	4.52E-30	TRUE	11.52799992	Cluster higher
A11_14	4	0.67059479	0.602757072	0.067837718	0.012462604	0.123212832	0.02007329	0.022868305	TRUE	2.324975128	Cluster higher
A11_14	5	0.697940675	0.602757072	0.095183604	0.01670868	0.173658527	0.024849442	0.027955622	TRUE	2.243735234	Cluster higher
A11_14	6	0.098604232	0.602757072	-0.50415284	-0.545915115	-0.462390565	1.54E-52	1.54E-51	TRUE	-15.25419434	Cluster lower
A11_15	1	0.846405968	0.666616906	0.179789062	0.145939232	0.213638892	1.70E-18	3.49E-18	TRUE	8.775336623	Cluster higher
A11_15	2	0.537500316	0.666616906	-0.12911659	-0.168011294	-0.090221886	2.97E-11	4.86E-11	TRUE	-6.648187843	Cluster lower
A11_15	3	0.937729231	0.666616906	0.271112325	0.239545259	0.30267939	9.31E-25	2.89E-24	TRUE	10.27313985	Cluster higher
A11_15	4	0.698871997	0.666616906	0.032255092	-0.021710566	0.086220749	0.251502791	0.269467276	FALSE	1.146706869	Cluster higher
A11_15	5	0.796273258	0.666616906	0.129656353	0.060392819	0.198919886	0.001461508	0.001730733	TRUE	3.182219947	Cluster higher
A11_15	6	0.151993603	0.666616906	-0.514623303	-0.562955829	-0.466290778	3.67E-57	4.13E-56	TRUE	-15.93421355	Cluster lower
A11_2	1	0.785861526	0.568515831	0.217345695	0.179693089	0.254998302	2.49E-23	7.22E-23	TRUE	9.951398794	Cluster higher
A11_2	2	0.401289365	0.568515831	-0.167226466	-0.206075247	-0.128377686	1.00E-16	1.84E-16	TRUE	-8.304441241	Cluster lower
A11_2	3	0.908149405	0.568515831	0.339633574	0.303412815	0.375854334	1.12E-33	6.70E-33	TRUE	12.09538927	Cluster higher
A11_2	4	0.680616856	0.568515831	0.112101025	0.057032871	0.16716918	1.46E-04	1.80E-04	TRUE	3.797507748	Cluster higher
A11_2	5	0.161620483	0.568515831	-0.406895348	-0.470921753	-0.342868943	5.09E-21	1.27E-20	TRUE	-9.407281053	Cluster lower
A11_2	6	0.19133325	0.568515831	-0.37718258	-0.429823314	-0.324541847	6.26E-30	2.68E-29	TRUE	-11.36487873	Cluster lower
A11_3	1	0.83758362	0.649702707	0.187880913	0.153365368	0.222396457	1.54E-19	3.46E-19	TRUE	9.042158215	Cluster higher
A11_3	2	0.532741101	0.649702707	-0.116961606	-0.155982613	-0.0779406	2.28E-09	3.60E-09	TRUE	-5.976709786	Cluster lower
A11_3	3	0.908074938	0.649702707	0.25837223	0.222521936	0.294222525	5.93E-22	1.52E-21	TRUE	9.630734328	Cluster higher
A11_3	4	0.776910616	0.649702707	0.127207909	0.077535545	0.176880273	6.76E-06	8.45E-06	TRUE	4.501192744	Cluster higher
A11_3	5	0.202673498	0.649702707	-0.44702921	-0.516228486	-0.377829933	2.11E-26	7.31E-26	TRUE	-10.63219948	Cluster lower

A11_3	6	0.296924309	0.649702707	-0.352778398	-0.412603306	-0.292953491	1.02E-27	3.83E-27	TRUE	-10.91091176	Cluster lower
A11_4	1	0.247047744	0.300070929	-0.053023185	-0.091398166	-0.014648203	0.009093043	0.010491973	TRUE	-2.608536065	Cluster lower
A11_4	2	0.248073834	0.300070929	-0.051997095	-0.086667041	-0.017327148	0.004382185	0.005122034	TRUE	-2.849254223	Cluster lower
A11_4	3	0.593150214	0.300070929	0.293079286	0.238094175	0.348064396	4.19E-27	1.51E-26	TRUE	10.78196986	Cluster higher
A11_4	4	0.305122749	0.300070929	0.00505182	-0.04891689	0.05902053	0.853905553	0.863499997	FALSE	0.184137546	Cluster higher
A11_4	5	0.485887096	0.300070929	0.185816167	0.101062802	0.270569532	3.76E-06	4.91E-06	TRUE	4.624092537	Cluster higher
A11_4	6	0.087609318	0.300070929	-0.21246161	-0.251962368	-0.172960852	1.19E-12	2.10E-12	TRUE	-7.106203952	Cluster lower
A11_5	1	0.523067818	0.514232004	0.008835815	-0.035047205	0.052718834	0.693233089	0.717137678	FALSE	0.394471332	Cluster higher
A11_5	2	0.487221504	0.514232004	-0.027010499	-0.066522962	0.012501963	0.180404827	0.195619692	FALSE	-1.339509613	Cluster lower
A11_5	3	0.833943133	0.514232004	0.31971113	0.27572563	0.36369663	5.64E-29	2.31E-28	TRUE	11.17126218	Cluster higher
A11_5	4	0.377310059	0.514232004	-0.136921945	-0.19396496	-0.07987893	4.72E-06	5.98E-06	TRUE	-4.576905348	Cluster lower
A11_5	5	0.764384572	0.514232004	0.250152569	0.177139527	0.32316561	8.08E-09	1.17E-08	TRUE	5.766783913	Cluster higher
A11_5	6	0.171474178	0.514232004	-0.342757826	-0.393574804	-0.291940848	6.40E-25	2.06E-24	TRUE	-10.30929125	Cluster lower
A11_6	1	0.096557075	0.335136115	-0.23857904	-0.268290067	-0.208868014	6.09E-32	3.04E-31	TRUE	-11.76252917	Cluster lower
A11_6	2	0.238707337	0.335136115	-0.096428778	-0.131057696	-0.06179986	2.42E-07	3.31E-07	TRUE	-5.16344738	Cluster lower
A11_6	3	0.860571882	0.335136115	0.525435767	0.484372299	0.566499235	1.50E-77	3.37E-76	TRUE	18.64081294	Cluster higher
A11_6	4	0.806332653	0.335136115	0.471196538	0.42375701	0.518636067	9.55E-59	1.23E-57	TRUE	16.16069369	Cluster higher
A11_6	5	0.099759093	0.335136115	-0.235377022	-0.288387449	-0.182366594	6.83E-09	1.01E-08	TRUE	-5.794947316	Cluster lower
A11_6	6	0.059070173	0.335136115	-0.276065942	-0.310758827	-0.241373057	2.93E-19	6.44E-19	TRUE	-8.971232302	Cluster lower
A11_7	1	0.047898089	0.306056662	-0.258158573	-0.282850412	-0.233466734	6.25E-40	4.68E-39	TRUE	-13.22557361	Cluster lower
A11_7	2	0.199434498	0.306056662	-0.106622164	-0.139474004	-0.073770325	3.87E-09	6.01E-09	TRUE	-5.88956662	Cluster lower
A11_7	3	0.860276184	0.306056662	0.554219522	0.513323712	0.595115332	3.66E-88	1.60E-86	TRUE	19.90528403	Cluster higher
A11_7	4	0.790222	0.306056662	0.484165338	0.435671025	0.53265965	9.84E-64	1.48E-62	TRUE	16.85377396	Cluster higher
A11_7	5	0.089427389	0.306056662	-0.216629273	-0.267265018	-0.165993528	4.51E-08	6.24E-08	TRUE	-5.469634323	Cluster lower
A11_7	6	0.045764295	0.306056662	-0.260292367	-0.291967842	-0.228616893	3.61E-18	7.21E-18	TRUE	-8.690608466	Cluster lower
A11_8	1	0.092009497	0.354592492	-0.262582995	-0.292066889	-0.233099101	2.74E-37	1.90E-36	TRUE	-12.75992764	Cluster lower
A11_8	2	0.244760675	0.354592492	-0.109831817	-0.144806079	-0.074857555	6.52E-09	9.79E-09	TRUE	-5.802744018	Cluster lower
A11_8	3	0.92017339	0.354592492	0.565580898	0.531300577	0.599861219	5.33E-88	1.60E-86	TRUE	19.88644447	Cluster higher
A11_8	4	0.870942761	0.354592492	0.516350269	0.474808724	0.557891813	8.48E-69	1.53E-67	TRUE	17.52981933	Cluster higher
A11_8	5	0.162985154	0.354592492	-0.191607338	-0.255632848	-0.127581828	3.47E-06	4.59E-06	TRUE	-4.640922411	Cluster lower
A11_8	6	0.045374785	0.354592492	-0.309217707	-0.341207036	-0.277228378	3.73E-23	1.05E-22	TRUE	-9.911057657	Cluster lower
A11_9	1	0.116213842	0.36222134	-0.246007498	-0.277486356	-0.214528639	2.52E-32	1.34E-31	TRUE	-11.83669444	Cluster lower
A11_9	2	0.271034538	0.36222134	-0.091186802	-0.127052936	-0.055320667	1.81E-06	2.43E-06	TRUE	-4.773712112	Cluster lower

A11_9	3	0.939574417	0.36222134	0.577353077	0.54585796	0.608848195	3.84E-91	3.45E-89	TRUE	20.24619093	Cluster higher
A11_9	4	0.797271575	0.36222134	0.435050236	0.38678081	0.483319662	2.04E-49	1.83E-48	TRUE	14.77783007	Cluster higher
A11_9	5	0.134288283	0.36222134	-0.227933056	-0.287505903	-0.16836021	3.81E-08	5.35E-08	TRUE	-5.499623213	Cluster lower
A11_9	6	0.057353421	0.36222134	-0.304867919	-0.339405063	-0.270330775	2.69E-22	7.11E-22	TRUE	-9.711754135	Cluster lower
A5_new_1	1	0.427490535	0.47290283	-0.045412296	-0.088952308	-0.001872284	0.042183613	0.056244817	FALSE	-2.031704175	Cluster lower
A5_new_1	2	0.543505818	0.47290283	0.070602987	0.03120403	0.110001945	4.62E-04	9.25E-04	TRUE	3.501607298	Cluster higher
A5_new_1	3	0.519502584	0.47290283	0.046599754	-0.009763214	0.102962721	0.105108548	0.132768692	FALSE	1.620576256	Cluster higher
A5_new_1	4	0.300245186	0.47290283	-0.172657644	-0.226969381	-0.118345907	6.77E-09	3.25E-08	TRUE	-5.796553269	Cluster lower
A5_new_1	5	0.69869794	0.47290283	0.225795109	0.147276001	0.304314217	2.00E-07	6.54E-07	TRUE	5.199715922	Cluster higher
A5_new_1	6	0.381729288	0.47290283	-0.091173543	-0.154685855	-0.02766123	0.006003532	0.009936881	TRUE	-2.747588411	Cluster lower
A5_new_2	1	0.701423564	0.565304146	0.136119419	0.095225342	0.177013495	6.38E-10	4.31E-09	TRUE	6.180717724	Cluster higher
A5_new_2	2	0.593343628	0.565304146	0.028039482	-0.010880184	0.066959149	0.159984789	0.187299265	FALSE	1.405122722	Cluster higher
A5_new_2	3	0.678829858	0.565304146	0.113525713	0.060441443	0.166609982	6.48E-05	1.48E-04	TRUE	3.99468458	Cluster higher
A5_new_2	4	0.371675271	0.565304146	-0.193628875	-0.250465157	-0.136792593	8.19E-11	9.82E-10	TRUE	-6.497122268	Cluster lower
A5_new_2	5	0.288716402	0.565304146	-0.276587743	-0.354164313	-0.199011174	1.56E-10	1.50E-09	TRUE	-6.39930913	Cluster lower
A5_new_2	6	0.376227255	0.565304146	-0.189076891	-0.252383843	-0.125769939	1.13E-08	4.92E-08	TRUE	-5.710206002	Cluster lower
A5_new_3	1	0.657724575	0.523563086	0.134161489	0.092031672	0.176291305	1.77E-09	9.46E-09	TRUE	6.017200441	Cluster higher
A5_new_3	2	0.533982567	0.523563086	0.010419481	-0.029027011	0.049865973	0.604895552	0.645221922	FALSE	0.517373361	Cluster higher
A5_new_3	3	0.61527864	0.523563086	0.091715554	0.036645615	0.146785493	0.001401638	0.002491802	TRUE	3.194313453	Cluster higher
A5_new_3	4	0.356795354	0.523563086	-0.166767732	-0.223219284	-0.110316181	2.48E-08	9.91E-08	TRUE	-5.574808218	Cluster lower
A5_new_3	5	0.297916643	0.523563086	-0.225646444	-0.303929739	-0.147363149	2.04E-07	6.54E-07	TRUE	-5.195316845	Cluster lower
A5_new_3	6	0.364746545	0.523563086	-0.158816541	-0.221804871	-0.095828211	1.80E-06	4.80E-06	TRUE	-4.774861795	Cluster lower
A5_new_4	1	0.428593355	0.372631923	0.055961432	0.012685849	0.099237014	0.010248169	0.015868132	TRUE	2.567341308	Cluster higher
A5_new_4	2	0.378437589	0.372631923	0.005805666	-0.032505355	0.044116688	0.766105732	0.782405854	FALSE	0.297472589	Cluster higher
A5_new_4	3	0.470292617	0.372631923	0.097660693	0.041560498	0.153760889	4.88E-04	9.36E-04	TRUE	3.487515412	Cluster higher
A5_new_4	4	0.349172869	0.372631923	-0.023459054	-0.079451974	0.032533866	0.416735721	0.454620786	FALSE	-0.812097442	Cluster lower
A5_new_4	5	0.115802001	0.372631923	-0.256829922	-0.313155451	-0.200504392	7.18E-10	4.31E-09	TRUE	-6.161998991	Cluster lower
A5_new_4	6	0.252696107	0.372631923	-0.119935816	-0.177212696	-0.062658936	1.76E-04	3.83E-04	TRUE	-3.751600496	Cluster lower
A5_new_5	1	0.575874343	0.462540505	0.113333838	0.069841111	0.156826564	4.21E-07	1.26E-06	TRUE	5.059357199	Cluster higher
A5_new_5	2	0.511970715	0.462540505	0.04943021	0.009940044	0.088920376	0.014119835	0.020537942	TRUE	2.454200183	Cluster higher
A5_new_5	3	0.521052527	0.462540505	0.058512022	0.002164463	0.11485958	0.041677238	0.056244817	FALSE	2.036728691	Cluster higher
A5_new_5	4	0.304976019	0.462540505	-0.157564486	-0.21207621	-0.103052762	1.16E-07	4.29E-07	TRUE	-5.29926564	Cluster lower
A5_new_5	5	0.094063559	0.462540505	-0.368476946	-0.420667694	-0.316286198	1.37E-17	6.59E-16	TRUE	-8.537451599	Cluster lower

**A5_new_5**	**6**	**0.342348449**	**0.462540505**	**-0.120192056**	**-0.182359555**	**-0.058024556**	**2.83E-04**	**5.91E-04**	**TRUE**	**-3.630038934**	**Cluster lower**
**A5_new_6**	**1**	**0.572315096**	**0.512818748**	**0.059496348**	**0.015944228**	**0.103048469**	**0.007858638**	**0.01257382**	**TRUE**	**2.658083679**	**Cluster higher**
A5_new_6	2	0.522096678	0.512818748	0.00927793	-0.030215932	0.048771792	0.645321988	0.673379466	FALSE	0.460270618	Cluster higher
**A5_new_6**	**3**	**0.595789579**	**0.512818748**	**0.082970832**	**0.027479347**	**0.138462316**	**0.003904109**	**0.006692758**	**TRUE**	**2.885807504**	**Cluster higher**
A5_new_6	4	0.517680824	0.512818748	0.004862076	-0.053737324	0.063461476	0.87084691	0.87084691	FALSE	0.162582826	Cluster higher
**A5_new_6**	**5**	**0.17007883**	**0.512818748**	**-0.342739917**	**-0.408020718**	**-0.277459116**	**3.03E-15**	**7.27E-14**	**TRUE**	**-7.88966037**	**Cluster lower**
**A5_new_6**	**6**	**0.405427331**	**0.512818748**	**-0.107391417**	**-0.171521528**	**-0.043261306**	**0.001245397**	**0.002299194**	**TRUE**	**-3.228273825**	**Cluster lower**
A5_new_8	1	0.278212171	0.307942109	-0.029729938	-0.069337006	0.00987713	0.148536396	0.178243675	FALSE	-1.444720548	Cluster lower
A5_new_8	2	0.347137037	0.307942109	0.039194928	0.00183993	0.076549926	0.036858351	0.052035319	FALSE	2.087329599	Cluster higher
**A5_new_8**	**3**	**0.433828842**	**0.307942109**	**0.125886733**	**0.070423231**	**0.181350235**	**2.93E-06**	**7.40E-06**	**TRUE**	**4.67585561**	**Cluster higher**
A5_new_8	4	0.258293296	0.307942109	-0.049648814	-0.101299854	0.002002227	0.070801513	0.091850612	FALSE	-1.806748427	Cluster lower
**A5_new_8**	**5**	**0.058645809**	**0.307942109**	**-0.24929063**	**-0.292250985**	**-0.206341615**	**3.10E-10**	**2.48E-09**	**TRUE**	**-6.293538126**	**Cluster lower**
A5_new_8	6	0.282821235	0.307942109	-0.025120874	-0.083985323	0.033743574	0.41245446	0.454620786	FALSE	-0.819581962	Cluster lower
**A5_new_9**	**1**	**0.432024137**	**0.376569788**	**0.055454348**	**0.012127063**	**0.098781633**	**0.011105026**	**0.016657539**	**TRUE**	**2.539376453**	**Cluster higher**
A5_new_9	2	0.40246037	0.376569788	0.025890582	-0.01276196	0.064543124	0.186667089	0.213333816	FALSE	1.320503138	Cluster higher
**A5_new_9**	**3**	**0.492836857**	**0.376569788**	**0.116267068**	**0.060070503**	**0.172463633**	**3.48E-05**	**8.34E-05**	**TRUE**	**4.139791212**	**Cluster higher**
A5_new_9	4	0.331566277	0.376569788	-0.045003511	-0.100394124	0.010387102	0.119563697	0.147155319	FALSE	-1.556607523	Cluster lower
**A5_new_9**	**5**	**0.072195299**	**0.376569788**	**-0.30437449**	**-0.351298962**	**-0.257450017**	**2.95E-13**	**4.73E-12**	**TRUE**	**-7.296466138**	**Cluster lower**
**A5_new_9**	**6**	**0.220229692**	**0.376569788**	**-0.156340097**	**-0.211272998**	**-0.101407195**	**1.02E-06**	**2.87E-06**	**TRUE**	**-4.888490906**	**Cluster lower**

Table S3 - Question codes and survey wording, alongside coding used for statistical analysis

This table provides a full list of question codes alongside their corresponding survey question wording. The coding applied to answers grouped for statistical testing is listed alongside each question.

Question code	Question wording	Coding for analysis
A5_new_	The following are reasons that people sometimes give for drinking alcohol. Thinking of all the times you drink, how often would you say that you drink for the following reasons?<br>-[A5_new_1] Drinking at home alone<br>-[A5_new_2] A small number of drinks at home with people in my household<br>-[A5_new_3] Several drinks at home with people in my household<br>-[A5_new_4] Getting together at your or someone else's house<br>-[A5_new_5] Going out for a meal<br>-[A5_new_6] Evening or night out with friends<br>-[A5_new_8] Going out for a couple of drinks in the afternoon<br>-[A5_new_9] Drinking at events	1 (Once a week or more), 2 (1-3 times a month) = positive; all other responses = negative
A11	How often, if at all, in the last year did you drink alcohol on occasions that are similar to the descriptions below?<br>- [A11_1] Because drinking is part of the fun with family or friends<br>-[A11_2] Because drinking adds a certain warmth to social occasions<br>-[A11_3] To celebrate a special occasion with friends<br>-[A11_4] To calm down when you are tense<br>-[A11_5] To help you unwind<br>-[A11_6] To make you more outgoing<br>-[A11_7] To overcome shyness<br>-[A11_8] Because you feel more self-confident and sure of yourself<br>-[A11_9] To put you at ease with people<br>-[A11_10] Because it is satisfying to have a high-quality drink<br>-[A11_11] Because it pairs well with food<br>-[A11_12] Because there are certain products you particularly enjoy<br>-[A11_13] Because you like the taste<br>-[A11_14] Because it makes you happy<br>-[A11_15] Because it gives you a pleasant feeling	3 (Half of the time), 4 (Most of the time), or 5 (Almost always/always) = positive; 1 (Almost never/never) or 2 (Some of the time) = negative
T1	How often during the last year have you found that you were not able to stop drinking once you had started?	3 (Monthly), 4 (Weekly), or 5 (Daily or almost daily) = positive; 1 (Never) or 2 (Less than monthly) = negative
T2	How often during the last year have you failed to do what was normally expected from you because of your drinking?	3 (Monthly), 4 (Weekly), or 5 (Daily or almost daily) = positive; 1 (Never) or 2 (Less than monthly) = negative
T3	How often during the last year have you needed an alcoholic drink in the morning to get yourself going after a heavy drinking session?	3 (Monthly), 4 (Weekly), or 5 (Daily or almost daily) = positive; 1 (Never) or 2 (Less than monthly) = negative
T4	How often during the last year have you had a feeling of guilt or remorse after drinking?	3 (Monthly), 4 (Weekly), or 5 (Daily or almost daily) = positive; 1 (Never) or 2 (Less than monthly) = negative
T5	How often during the last year have you been unable to remember what happened the night before because you had been drinking?	3 (Monthly), 4 (Weekly), or 5 (Daily or almost daily) = positive; 1 (Never) or 2 (Less than monthly) = negative
T6	Have you or somebody else been injured as a result of your drinking?	3 (Yes, during the last year) = positive; 1 (No) and 2 (Yes, but not in the last year) = negative
T7	Has a relative or friend, doctor or other health worker been concerned about your drinking or suggested that you cut down?	3 (Yes, during the last year) = positive; 1 (No) and 2 (Yes, but not in the last year) = negative
A1	How often do you have a drink containing alcohol?	1 (6 or more times a week) and 2 (4 or 5 times a week) = positive; all other responses = negative
Q6B	How many units of alcohol do you drink on a typical day when drinking?	3 (5 or 6), 4 (7 to 9), 5 (10 or more) = positive; All other responses = negative
Q6C	How often have you had 6 units or more on a single occasion in the last year? Again, please use the above guidance to complete your answer.	4 (weekly), 5 (daily or almost daily) = positive; All other responses = negative
MH_5_1	How often, if ever, do you feel that you lack companionship	1 (Often/always), 2 (Some of the time), or 3 (Occassionally) = positive; 4 (Hardly ever) or 5 (Never) = negative
MH_5_2	How often, if ever, do you feel left out	1 (Often/always), 2 (Some of the time), or 3 (Occassionally) = positive; 4 (Hardly ever) or 5 (Never) = negative
MH_5_3	How often, if ever, do you feel isolated from others	1 (Often/always), 2 (Some of the time), or 3 (Occassionally) = positive; 4 (Hardly ever) or 5 (Never) = negative
MH_5_4	How often, if ever, do you feel lonely	1 (Often/always), 2 (Some of the time), or 3 (Occassionally) = positive; 4 (Hardly ever) or 5 (Never) = negative
A7	Thinking about your alcohol consumption, how likely or not do you think it is that you will have increased health problems in the future if you continue to drink at your current level?	1 (Very likely), 2 (Fairly likely) = positive; All other responses = negative
DV3_1	Thinking about the last 12 months, how often, if at all, have you experienced pressure to drink alcoholic drinks even if others know you aren't drinking	1 (Always), 2 (Often), or 3 (Sometimes) = positive; All other responses = negative

DV3_2	Thinking about the last 12 months, how often, if at all, have you experienced being asked to explain or justify why you aren't drinking alcohol	1 (Always), 2 (Often), or 3 (Sometimes) = positive; All other responses = negative
DV3_3	Thinking about the last 12 months, how often, if at all, have you experienced trying to hide that you are not drinking alcohol from others	1 (Always), 2 (Often), or 3 (Sometimes) = positive; All other responses = negative
DV3_4	Thinking about the last 12 months, how often, if at all, have you experienced feeling like an outsider in a situation where others are drinking alcohol	1 (Always), 2 (Often), or 3 (Sometimes) = positive; All other responses = negative
T12	Others concerned or suggested cutting down due to drinking	1 (Yes - I successfully reduced my alcohol consumption), 2 (Yes - I successfully reduced my alcohol consumption for a bit, but have returned to typical levels of drinking), or 3 (Yes - I tried to reduce my alcohol consumption but wasn't successful) = positive; 4 (No) = negative
East_of_England	Location: East (England)	N/A - (1 = Yes)
NET_Midlands	Location: Midlands (England)	N/A - (1 = Yes)
NET_North	Location: North (England)	N/A - (1 = Yes)
NET_South	Location: South (England)	N/A - (1 = Yes)
Northern_Ireland	Location: Northern Ireland	N/A - (1 = Yes)
Scotland	Location: Scotland	N/A - (1 = Yes)
Wales	Location: Wales	N/A - (1 = Yes)
age_cross_18.34	Age 18–34	N/A - (1 = Yes)
age_cross_35.54	Age 35.54	N/A - (1 = Yes)
age_cross_55_or_above	Age 55+	N/A - (1 = Yes)
ethnicity_Asian	Ethnicity: Asian	N/A - (1 = Yes)
ethnicity_Black	Ethnicity: Black	N/A - (1 = Yes)
ethnicity_Mixed	Ethnicity: Mixed	N/A - (1 = Yes)
ethnicity_NET_ethnic_minority	Ethnicity: Ethnic minority	N/A - (1 = Yes)
ethnicity_White	Ethnicity: White	N/A - (1 = Yes)
parent_non_parent	Non-parent	N/A - (1 = Yes)
parent_to_child_over_18	Parent to child over 18	N/A - (1 = Yes)
parent_to_child_u18	Parent to child under 18	N/A - (1 = Yes)
IMD_decile_2020_1_to_3	IMD decile 2020 1 to 3	N/A - (1 = Yes)
IMD_decile_2020_4_to_7	IMD decile 2020 4 to 7	N/A - (1 = Yes)
IMD_decile_2020_8_to_10	IMD decile 2020 8 to 10	N/A - (1 = Yes)
social_grade_ABC1	Social grade ABC1	N/A - (1 = Yes)
social_grade_C2DE	Social grade C2DE	N/A - (1 = Yes)
